# Mycoplasma infection drives EGFR-TKI resistance through convergent transcriptomic reprogramming in non-small cell lung cancer

**DOI:** 10.1016/j.isci.2026.116999

**Published:** 2026-07-31

**Authors:** Bing Wang, Valentina Donati, Alessandro Gregori, Alessandro Leonetti, Aldo Pastore, Dongmei Deng, Elisa Giovannetti

**Affiliations:** 1 Department of Medical Oncology, Cancer Center Amsterdam, VU University Medical Center, Amsterdam, the Netherlands; 2 Cancer Biology and Immunology, Cancer Center Amsterdam, Amsterdam, The Netherlands; 3 Unit of Pathological Anatomy 2, Azienda Ospedaliero-Universitaria Pisana, Pisa, Italy; 4 Medical Oncology Unit, University Hospital of Parma, Parma, Italy; 5 Cancer Pharmacology Lab, Fondazione Pisana per la Scienza, San Giuliano, Pisa, Italy; 6 Department of Preventive Dentistry, Academic Centre for Dentistry Amsterdam (ACTA), University of Amsterdam and Vrije Universiteit Amsterdam, Amsterdam, the Netherlands

**Keywords:** non-small cell lung cancer, *Mycoplasma*, EGFR-TKI resistance, transcriptomics, machine learning

## Abstract

Epidermal growth factor receptor (EGFR) mutations are an oncogenic driver in non-small cell lung cancer (NSCLC), but resistance to EGFR tyrosine kinase inhibitors (TKIs) remains a major challenge. Here, we show that mycoplasma infection sustains ERK, AKT, and NF-κB signaling; impairs receptor internalization; and reduces osimertinib sensitivity in EGFR-mutant NSCLC cells. Antibiotic-mediated clearance of *Mycoplasma* partially restored drug sensitivity, establishing a causal infection-resistance link. Transcriptomic analysis identified converging pathways related to mycoplasma infection and drug resistance, including gene sets enriched for inhibition of receptor internalization and clathrin downregulation at the protein level. Combined inhibition of mTOR and LDHA improved growth suppression in mycoplasma-positive cells. Clinically, mycoplasma infection and higher LDHA expression were associated with poor overall survival of EGFR-TKI-treated patients. These findings establish mycoplasma infection as a causal, non-genetic contributor to EGFR-TKI resistance and support a hierarchical therapeutic strategy combining antibiotic eradication with mTOR and LDHA inhibition.

## Highlights

• Mycoplasma infection enhances EGFR-TKI resistance in EGFR-mutant NSCLC
• Transcriptomic profiling identifies convergent infection and drug resistance programs
• *Mycoplasma* impairs EGFR trafficking and sustains downstream signaling
• Targeting mTOR and LDHA overcomes resistance with clinical prognostic support

## Graphical abstract

**Figure:**
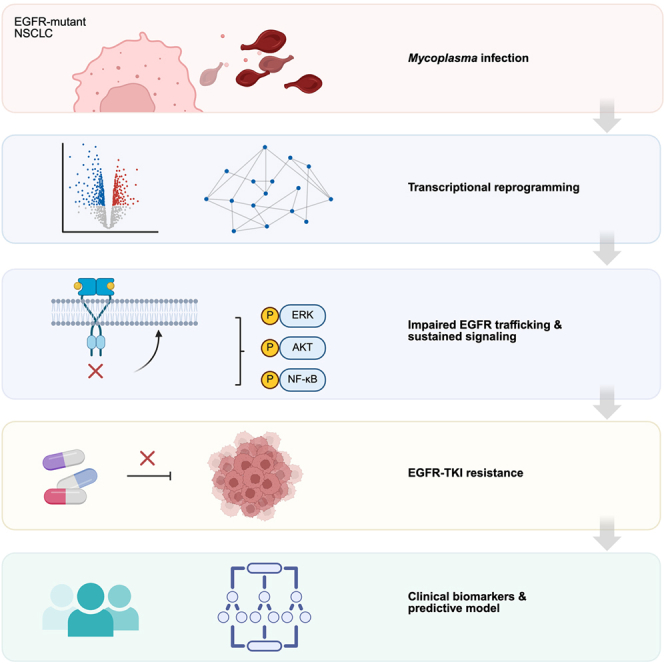

## Teaser

Microbiology; Cell biology; Cancer

## Introduction

Lung cancer is one of the leading causes of cancer-related deaths, accounting for approximately 20.8% of all cancer mortalities worldwide.^1^ The 5-year survival rate is only 25%.^2^ Non-small cell lung cancer (NSCLC) constitutes 80%–85% of all lung cancer cases and is molecularly heterogeneous. The identification of oncogenic drivers, particularly alterations in the epidermal growth factor receptor (EGFR), has revolutionized the treatment of NSCLC.^3^ EGFR mutations occur in 8%–16% of western patients and up to 50% of East Asian patients with NSCLC.^4^ Exon 19 deletions and exon 21 L858R substitutions are the most common sensitizing alterations, leading to constitutive activation of MAPK/ERK and PI3K/AKT signaling.^5^^,^^6^

EGFR-targeted tyrosine kinase inhibitors (TKIs) have significantly improved outcomes compared with chemotherapy in EGFR-mutant patients.^7^^,^^8^^,^^9^ The clinical success of TKIs has progressed through three generations: first-generation reversible inhibitors (gefitinib and erlotinib), second-generation irreversible pan-HER inhibitors (afatinib and dacomitinib), and third-generation mutation-selective inhibitors (osimertinib).^10^^,^^11^^,^^12^ Osimertinib has become the standard first-line treatment for advanced EGFR-mutant NSCLC by selectively inhibiting both EGFR-TKI-sensitizing and T790M resistance mutation.^13^

Despite these advances, acquired resistance emerges in approximately 50% of patients within 10–15 months. Resistance mechanisms are complex and include on-target changes such as secondary EGFR mutations (T790M in first/second-generation TKI resistance,^14^^,^^15^ C797S in osimertinib resistance),^16^^,^^17^ off-target bypass signaling through alternative receptor tyrosine kinases (MET amplification, HER2 amplification, and AXL upregulation),^18^^,^^19^^,^^20^ activation of downstream pathways (KRAS mutations, PIK3CA mutations, and loss of PTEN),^21^^,^^22^^,^^23^ and changes in phenotype, such as epithelial-mesenchymal transition (EMT) and histological transformation to small cell lung cancer.^24^^,^^25^^,^^26^ These various resistance mechanisms have prompted the creation of combination therapies and next-generation TKIs.^27^^,^^28^ However, a substantial percentage of resistance cases remain mechanistically unresolved.

In recent years, growing evidence suggests that the tumor microenvironment, including microbial factors, may affect how well treatments work and how resistance develops.^29^ Recent seminal studies have illustrated that intratumoral bacteria can directly metabolize and inactivate chemotherapeutic agents, with Geller et al. demonstrating that bacterial cytidine deaminase (CDA) can degrade gemcitabine in colorectal and pancreatic cancer models.^30^ Beyond direct drug metabolism, bacterial infection can modulate host cell signaling, immune responses, and treatment outcomes.^31^

*Mycoplasma* species are cell-wall-deficient bacteria capable of establishing persistent, often asymptomatic, infections in human tissues.^32^ These organisms are often detected in lung cancer patients and cell cultures, but their role in therapeutic resistance remains poorly characterized.^33^ Prior research has shown that elevated *Mycoplasma hyorhinis* infection is associated with significantly reduced progression-free survival, with higher infection levels correlating with more advanced disease.^34^ *M. hyorhinis* was also strongly linked to TKI resistance, a phenomenon attributed to its potential ability to hydrolyze TKI molecules. However, osimertinib lacks the key chemical features, specifically the sugar moiety and free amino group, required for CDA-mediated deamination, making this mechanism unlikely. Therefore, the molecular mechanisms linking mycoplasma exposure to EGFR-TKI resistance and their clinical significance remain insufficiently understood.

This study aims to investigate the mechanisms underlying mycoplasma-mediated resistance to EGFR-TKIs by using bioinformatics analyses along with biological and pharmacological validation.

Specifically, by integrating functional assays, genome-wide transcriptomic profiling, and machine learning approaches, we dissected how mycoplasma infection alters EGFR signaling dynamics. We demonstrated that mycoplasma-positive (M+) cells activate many of the same pathways seen in drug-resistant models. This unified convergence suggests that infection may “prime” cells for resistance acquisition, positioning *Mycoplasma* as an important factor contributing to clinical TKI failure.

## Results

### Impact of mycoplasma infection on EGFR trafficking, signaling, and TKI sensitivity

**Figure 1:**
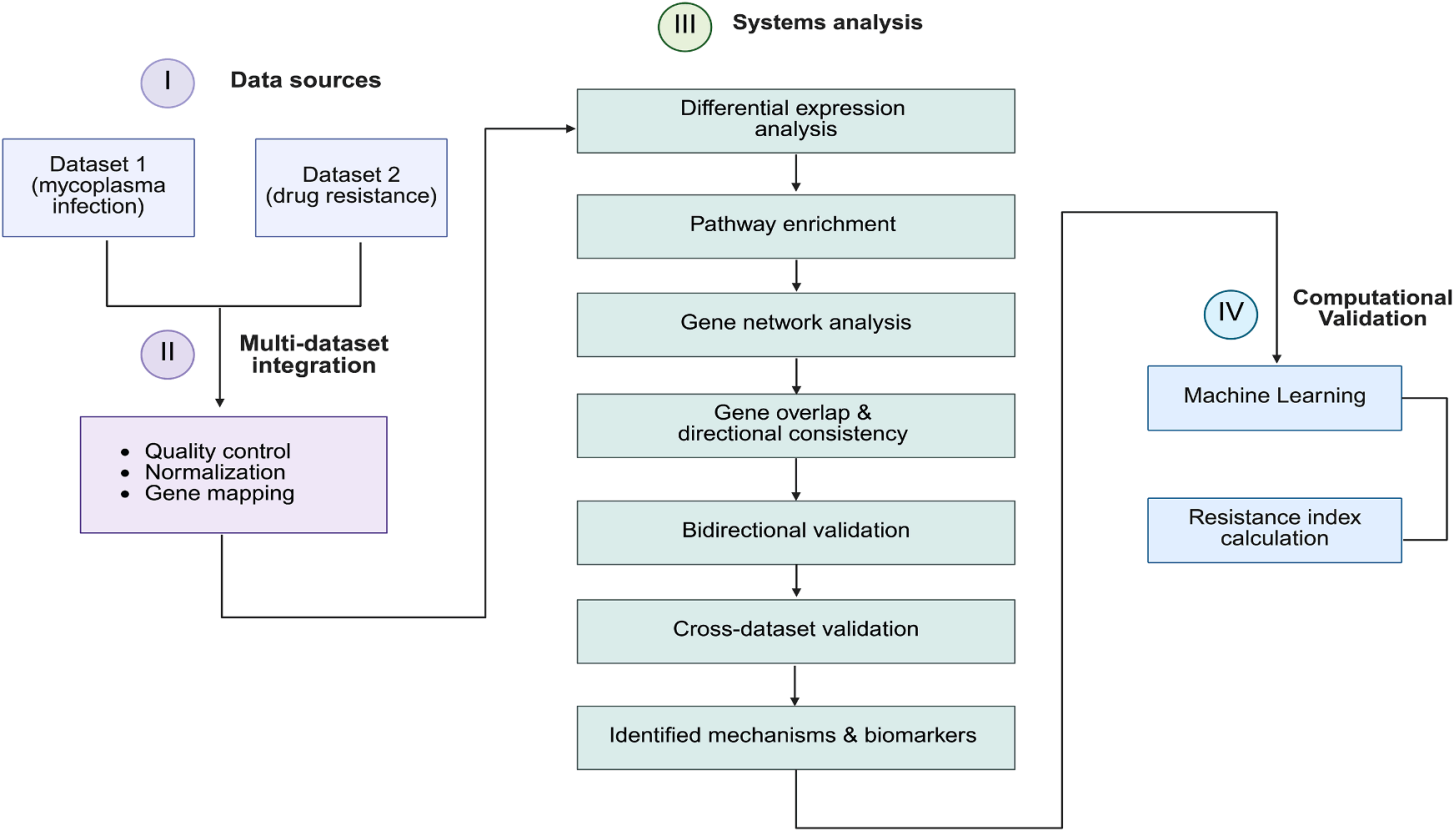
Overview of analytical workflow

**Figure 2:**
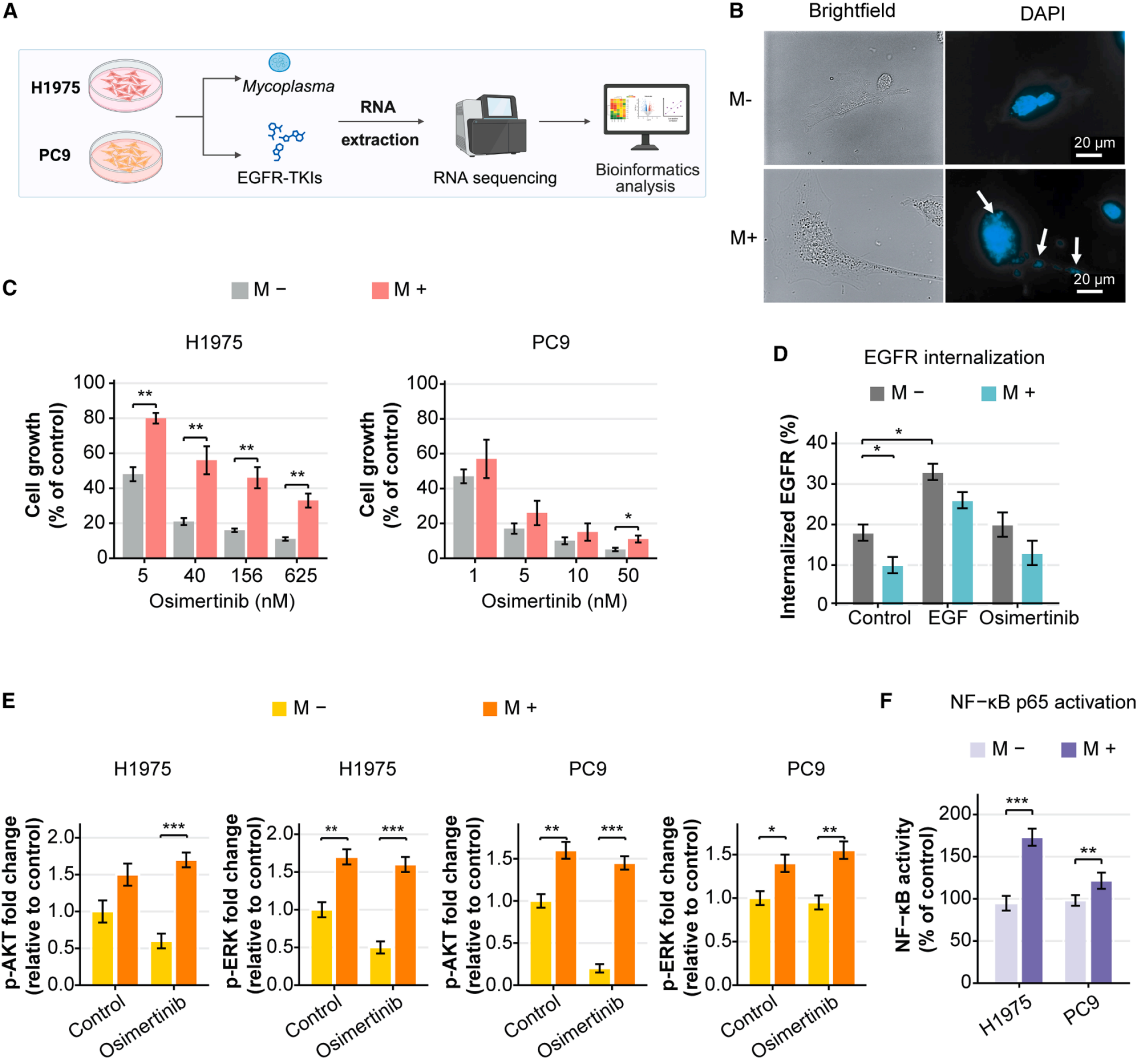
Functional validation confirms mycoplasma-induced TKI resistance (A) Workflow of RNA-seq and bioinformatics analysis in H1975 and PC9 cells with or without mycoplasma infection. (B) *Mycoplasma* visualization via immunofluorescence. DAPI staining highlights infected cells (white arrows). Scale bars, 20 μm. (C) Growth inhibition curves show osimertinib sensitivity in mycoplasma-positive (M+) and mycoplasma-negative (M-) H1975 and PC9 cells. (D) Mycoplasma infection impairs EGFR internalization. (E) ELISA quantification of p-AKT and p-ERK levels in H1975 and PC9 M- and M+ cells with or without osimertinib treatment. (F) NF-κB ELISA quantification in M+ and M- H1975 and PC9 cells. All data are presented as mean ± SEM (standard error of the mean); ∗*p* < 0.05, ∗∗*p* < 0.01, ∗∗∗*p* < 0.001.

An overview of the complete analytical workflow is presented in Figure 1. Figure 2A summarizes the overall experimental workflow used to investigate the impact of mycoplasma infection on EGFR signaling and TKI response in EGFR-mutant NSCLC cells. We analyzed EGFR trafficking and downstream signaling in cells infected with *Mycoplasma*. Morphological analysis under the microscope did not show relevant changes in the structure of the cells upon mycoplasma infection. However, DAPI staining confirmed mycoplasma contamination, revealing characteristic perinuclear fluorescence and bacterial aggregates in infected cells (Figure 2B).

Next, we performed dose-response viability assays across a relevant concentration range of osimertinib (Figure 2C). In H1975 and PC9 cells, M+ conditions consistently showed higher relative cell growth compared with mycoplasma-negative (M-) controls. The dramatic shifts indicate reduced sensitivity to EGFR-TKI treatment and confirmed that mycoplasma infection substantially impairs EGFR-TKI efficacy across multiple mutational contexts. Quantitative EGFR internalization assays shown in Figure 2D demonstrate significant disparities between M+ and M- conditions. Under basal conditions, H1975 M- cells showed 20% EGFR internalization, confirming the normal receptor trafficking kinetics.^35^ In contrast, infected cells showed a lower rate of internalization (5%–10%; *p* < 0.05). Epidermal growth factor (EGF) stimulation eliminated this disparity, demonstrating that *Mycoplasma* specifically interferes with constitutive rather than ligand-induced internalization.^36^ Confocal microscopy further delineated this trafficking defect. In mycoplasma-infected cells, phosphorylated EGFR showed reduced colocalization with the lysosomal marker LAMP-3 and the early endosomal marker EEA1 (Figures S1A and S1B). Figure S1C shows the dose-response assays for afatinib, a second-generation EGFR-TKI. M+ cells displayed reduced sensitivity, indicating that resistance is not restricted to third-generation inhibitors.

Genomic analyses showed that mycoplasma-infected cells retained their original mutations without acquiring additional EGFR alterations. Specifically, H1975 cells harbor the EGFR exon 21 L858R mutation in *cis* with the T790M mutation in exon 20, whereas PC9 cells carry an E746–A750 deletion in exon 19 of the EGFR gene (c.2235_2249del15). RT-qPCR analysis revealed similar EGFR mRNA levels in both M+ and M- cells within the H1975 and PC9 cell lines, irrespective of osimertinib treatment (Figure S2). These results showed no significant changes in EGFR transcripts, suggesting that the phenotypes we saw are caused by post-translational processes rather than transcriptional changes at the EGFR level.^22^ Quantification of phosphoproteins by ELISA provides compelling evidence of enhanced signaling in mycoplasma-infected cells. Osimertinib treatment markedly decreased phosphoprotein levels in M- cells but showed limited effectiveness in M+ cells. In particular, in H1975 cells, mycoplasma infection significantly elevated phospho-AKT (*p* < 0.01) and phospho-ERK (*p* < 0.001) levels in basal conditions (Figure 2E). Compared with uninfected controls, M+ H1975 and PC9 cells displayed elevated NF-κB activity, as measured by ELISA (Figure 2F), supporting activation of inflammatory pathways identified by transcriptomic enrichment analyses of TNF and IL-17 signaling.

To further investigate the molecular basis of impaired EGFR internalization, we performed gene set enrichment analysis (GSEA) using Gene Ontology (GO) Biological Process terms (Figure S4). “Regulation of receptor internalization” and “receptor internalization” gene sets were negatively enriched in mycoplasma-infected cells in both H1975 and PC9 models (Figure S4A). Of particular mechanistic interest, it has been shown that CBL and CBLB mediate EGF-induced EGFR ubiquitination and are required for clathrin-mediated EGFR internalization.^35^ Those targets are among the leading-edge genes driving negative enrichment in both cell lines. These transcriptomic findings provide a molecular substrate for the observed EGFR internalization phenotype. The observed downregulation of endocytic regulators may result from either direct effect of mycoplasma-derived membrane components or indirect modulation through infection-induced NF-κB signaling, highlighting important mechanistic avenues for future biomedical studies.

This analysis confirmed downregulation of clathrin, a key endocytic component at the protein level in mycoplasma-infected cells (Figure S4B), consistent with the GSEA pathway findings. Together, the GSEA and the ELISA data provide multi-level convergent evidence of suppression of the endocytic machinery in mycoplasma-infected cells.

General inflammatory activation was modeled by exposing M- cells to lipopolysaccharide (LPS) before osimertinib treatment. LPS stimulation at 0.1, 1, and 10 μg/mL did not significantly alter osimertinib sensitivity in either H1975 or PC9 cells at any concentration tested (*p* > 0.05 compared with unstimulated controls, Figure S5). Both models failed to significantly affect drug sensitivity, suggesting that generalized inflammatory signaling alone is insufficient to reproduce the resistant phenotype.

### Comprehensive transcriptomic landscapes reveal mycoplasma-induced changes

**Figure 3:**
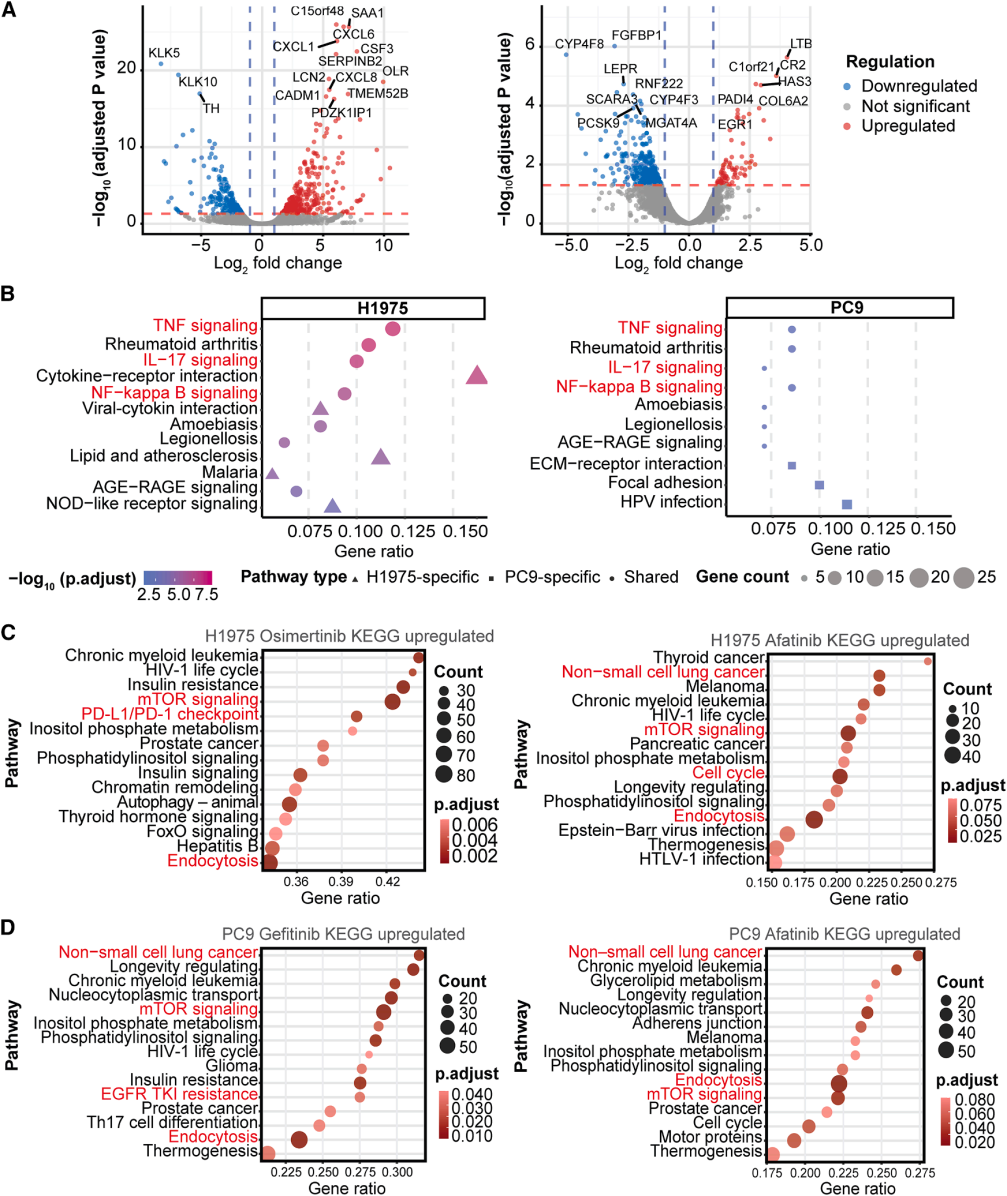
Transcriptomic profiling in mycoplasma-infected and drug-resistant cells (A) Volcano plots showing differentially expressed genes (DEGs) induced by mycoplasma infection in H1975 and PC9. Significantly upregulated and downregulated genes are highlighted; the top dysregulated genes are labeled. (B) KEGG pathway enrichment of DEGs in H1975 and PC9. Pathways are categorized as shared or cell-line specific. (C) Upregulated KEGG pathways in H1975 (osimertinib and afatinib) cells under mycoplasma infection. (D) Upregulated KEGG pathways in PC9 (gefitinib and afatinib) cells under mycoplasma infection. Gene ratio = number of DEGs in pathway/total number of genes in pathway. Statistical significance was assessed using the hypergeometric test with the Benjamini-Hochberg correction.

We developed a transcriptomic analysis pipeline to integrate the mycoplasma infection profiles and drug resistance signatures, depicted in Figure 1. Using the STAR aligner, raw FASTQ files were aligned to GRCh38.^37^ We used featureCounts to count reads at the gene level and DESeq2 to normalize the gene counts.^38^ Quality control filtering removed genes with low counts (<10 reads).^39^ The processed count matrix from both databases included both infection states and drug resistance variants. We used DESeq2 on each dataset separately for differential expression analysis, using the same threshold: an adjusted *p* value < 0.05. We identified 532 differentially expressed genes (DEGs) in H1975 cells (313 upregulated and 219 downregulated) and 425 DEGs in PC9 cells (79 upregulated and 346 downregulated) when comparing drug-resistant variants to wild-type cells (Figure 3A).

The volcano plot visualization revealed cell-line-specific responses to mycoplasma infection. H1975 cells, showed a significant increase in the levels of inflammatory mediators (CXCL8 and CXCL5) known to promote tumor progression and resistance,^40^ metabolic regulators (LDHA, IGBP3, and C15orf48),^41^ and signaling molecules (SRPBP3, CADM1, and TMEM52B). Moreover, a decrease was displayed for genes such as KLK5, IL1A10, and VT11 (Figure 3A). PC9 cells exhibited a profile characterized by the upregulation of similar metabolic regulators and stress response genes (FGFBP1, CYP4F3, and SCARB1) but also the downregulation of extracellular matrix (ECM) components (COL6A2 and PADI4), cell adhesion molecules (LTB, CtoC21, and LEPR). These latter distinct transcriptional changes indicate that mycoplasma infection leads to some cell-type-specific alterations.

We conducted a thorough pathway enrichment analysis utilizing GO, Kyoto Encyclopedia of Genes and Genomes (KEGG), Reactome, and Hallmark databases to elucidate the biological processes driving transcriptional shifts.^42^^,^^43^^,^^44^^,^^45^ Differential expression analysis showed unique transcriptomic signatures in each cell line. H1975 cells exhibited the most enriched inflammatory pathways, including TNF signaling, IL-17 signaling, rheumatoid arthritis, and cytokine-receptor interaction (Figure 3B). Other enriched pathways included NF-κB signaling, viral-cytokine interaction, malaria, and the AGE-RAGE signaling pathway.^46^^,^^47^ Together, these pathways suggest that responses to bacterial infection are largely mediated by immune-related signaling pathways.

In PC9 cells, pathway enrichment analysis revealed overlapping yet distinct patterns. While inflammatory pathways, including TNF, IL-17, and NF-κB signaling, were similarly activated, PC9 cells displayed additional enrichment in pathways associated with ECM remodeling and cell-matrix interactions, such as the AGE-RAGE signaling pathway, ECM-receptor interaction, and focal adhesion pathways (Figure 3B). These findings suggest that, beyond canonical inflammatory responses, mycoplasma infection may also modulate the tumor-microenvironment through pathways influencing extracellular architecture and cell adhesion.^48^^,^^49^ The disparity may be attributable to differences in the EGFR mutation context (L858R + T790M in H1975 versus an exon 19 deletion in PC9 cells).^50^^,^^51^

After identifying mycoplasma-induced pathways, we examined pathway enrichment in drug-resistant cell-line variants to determine whether any overlap existed. Multiple pathway analyses of drug-resistant cells showed strong similarities to signatures caused by *Mycoplasma* (Figures 3C, 3D, and S3). For example, in H1975 osimertinib-resistant cells, the most upregulated pathways included chronic myeloid leukemia, insulin resistance, the mTOR signaling pathway, PD-L1 expression and the PD-1 checkpoint pathway, inositol phosphate metabolism, ATP-dependent chromatin remodeling, FoxO signaling pathway, and endocytosis (Figure 3C, left). These pathways converge to control growth signaling, metabolic adaptation, and membrane trafficking, all of which are essential for TKI resistance.^52^^,^^53^ H1975 afatinib-resistant cells showed enrichment in different cancers, including NSCLC, cell cycle regulation, epithelial cell signaling during *Helicobacter pylori* infection, and human T cell leukemia virus infection (Figure 3C, right). The prominence of cancer-type pathways suggests the activation of pan-cancer survival mechanisms during the development of resistance.^54^^,^^55^

Similarly, in PC9 gefitinib-resistant cells, enrichment was observed across pathways relevant to tumor progression and therapeutic escape, including NSCLC, longevity regulation, mTOR signaling, insulin resistance, EGFR-TKI resistance, and Th17 cell differentiation (Figure 3D, left). The enrichment in the EGFR-TKI resistance pathway directly verifies the known mechanism in this model. PC9 afatinib-resistant cells exhibited a similar pathway with additional activation of adherent junction and longevity regulating pathways (Figure 3D, right). Of note, the enrichment of the longevity pathway hints at potential metabolic reprogramming toward resistance and survival.

It is important to note that some pathways were present in both drug resistance conditions (Figures 3C and 3D) and mycoplasma infection (Figure 3B). Remarkably, the mTOR signaling pathway was significantly upregulated in mycoplasma-infected cells and in H1975 osimertinib-resistant, PC9 gefitinib-resistant, and PC9 afatinib-resistant cells, underscoring its pivotal function as a convergent resistance mechanism.^56^ Similarly, endocytosis pathways appeared in all contexts of drug resistance and correspond with the influence of *Mycoplasma* on EGFR trafficking.^35^ In addition, metabolic pathways for phosphatidylinositol and inositol phosphate were universally enriched across all resistance conditions. This is consistent with the activation of the PI3K/AKT pathway observed in cells infected with *Mycoplasma*.^57^ The persistent activation of inflammatory pathways in both mycoplasma infection and drug resistance indicates that chronic inflammation may facilitate resistance mechanisms.^47^^,^^58^

**Figure 4:**
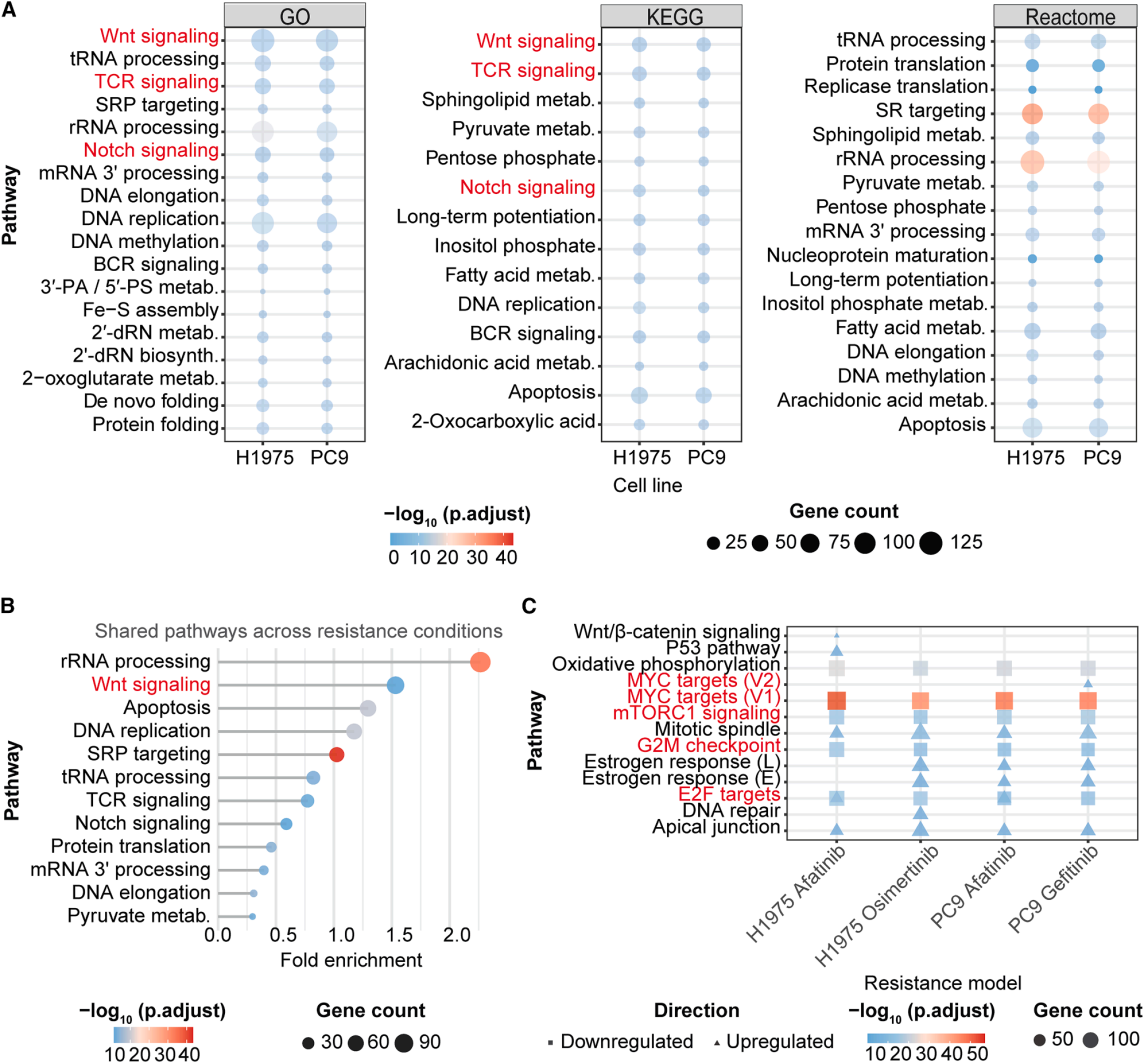
Cross-model pathway comparison (A) Pathway enrichment analysis across GO, KEGG, and Reactome databases in H1975 and PC9 cells. The number of conditions in which each pathway is enriched is displayed. (B) Core pathways are shared across all resistance conditions. Dot size indicates gene count and color represents significance (-log_10_ adjusted *p*value). (C) Hallmark pathways were consistently activated across four resistance models (H1975 afatinib, H1975 osimertinib, PC9 afatinib, and PC9 gefitinib). Shape denotes directionality (upregulated or downregulated), dot size represents gene count, and color indicates significance (-log_10_ adjusted *p*value).

Prompted by these results, we conducted cross-database enrichment analysis (Figure 4A) to systematically identify pathways that are common to both mycoplasma infection and drug resistance. The multi-database approach reduces annotation bias and justifies pathway identification during analyses. When we reviewed the GO, KEGG, and Reactome databases, we found both conserved and cell-line-specific pathway responses. In both H1975 and PC9 cells, mycoplasma infection enhanced fundamental cellular processes, including Wnt signaling and Notch signaling pathways.^59^^,^^60^ A comparative analysis of pathway sets identified processes enriched across multiple annotation sources. Wnt signaling was present in the GO, KEGG, and for both cell lines, signifying substantial enrichment. T cell receptor signaling, Notch signaling, DNA replication, and B cell receptor signaling also showed cross-dataset enrichment (Figure 4A).^61^ Fold enrichment analysis across resistance conditions disclosed pathways common to mycoplasma infection and drug resistance (Figure 4B). The most strongly enriched shared pathways were rRNA processing (fold enrichment approximately 2.0 and gene count >90), reflecting increased protein synthesis capacity of proliferating cancer cells.^62^^,^^63^ Other shared pathways included Wnt signaling, apoptosis, DNA replication, SRP co-translational targeting, and tRNA processing. Also, other pathways were shared between the groups (Figure 4B). Collectively, crossing conservation implies an initial cellular reprogramming process, such as biosynthetic capacity, cell cycle control, and metabolic rewiring.^41^

In addition, a hallmark GSEA across four drug resistance conditions revealed an ongoing oncogenic program, similar to the enrichment that was observed in mycoplasma-induced pathways (Figure 4C). MYC (V1 and V2) targets showed significant upregulation across all resistance conditions (H1975 afatinib, H1975 osimertinib, PC9 afatinib, and PC9 gefitinib) with the highest enrichment observed in H1975 afatinib-resistant cells. MYC is a master transcriptional regulator that controls cell growth, metabolism, and proliferation. It is frequently activated in cancer, where its dysregulation contributes to therapy, resistance, and impedes treatment efficacy.^64^ mTORC1 signaling was consistently upregulated across conditions, reflecting its enrichment in mycoplasma-infected cells and corroborating its central role as a convergent resistance mechanism.^56^ The G2M checkpoint pathway aligned moderate upregulation under resistance conditions, denoting cell cycle dysregulation (Figure 4C) in therapy-resistant states. In contrast, E2F targets, DNA repair, and apical junction pathways displayed comparatively minor enrichment. Particularly, E2F is a transcription factor that regulates cell cycle progression and DNA replication, contributing to therapeutic resistance.^65^ Oxidative phosphorylation and Wnt-β-catenin signaling were bidirectionally regulated, with the extent of regulation depending on the cell line and TKIs.^59^^,^^66^ The uniformity of MYC targets, mTORC1 signaling, and G2M checkpoint enrichment in both mycoplasma infection (inflammatory/metabolic pathways) and drug resistance (proliferative pathways) points to an important convergence on proliferative and survival mechanisms.

### Gene overlap analysis uncovers significant convergence with drug resistance mechanisms

**Figure 5:**
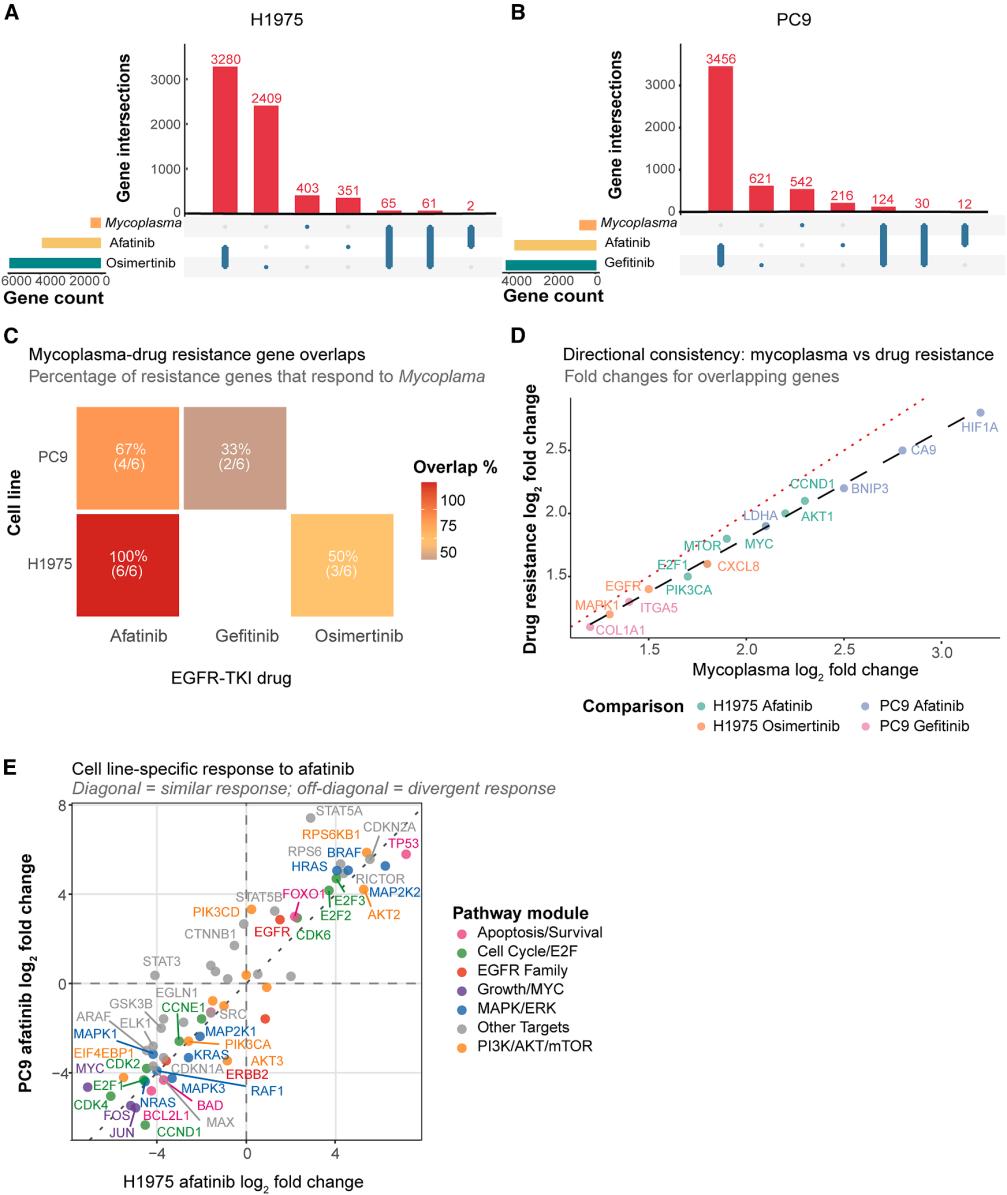
Convergent transcriptional responses link mycoplasma exposure to EGFR-TKI resistance (A and B) UpSet plots showing the gene intersection between mycoplasma infection and EGFR-TKI resistance in H1975 and PC9. Bar heights indicate the number of shared genes and horizontal bars (in colors) represent the number of genes per condition. (C) Proportion of drug-resistance genes that respond to *Mycoplasma*, quantified by each cell line and EGFR-TKI. (D) Directional consistency analysis of overlapping genes. Each point denotes an overlapping gene, with greater proximity to the diagonal indicating greater overlap. (E) Comparison of EGFR pathway response to afatinib between H1975 and PC9, classified by pathway module. Genes near the diagonal exhibit shared regulation, whereas off-diagonal genes exhibit cell-line-specific divergence.

Following the findings of pathway-level convergence between mycoplasma infection and drug resistance, we identified gene-level overlap to clarify specific molecular mediators. The UpSet plot analysis showed how big the transcriptional changes were between conditions (Figures 5A and 5B). Mycoplasma infection in H1975 cells altered more than 2,000 genes, indicating the most extensive transcriptional response. The mycoplasma infection exhibited significant overlap with afatinib and osimertinib resistance, as demonstrated by gene intersections (Figure 5A). The alteration in resistance to afatinib/gefitinib involves approximately 1,000 genes. The extent of transcriptional modifications indicates infection-related initiation, with extensive cellular reprogramming in resistance-related pathways. Among the overlapped genes, specific genes were curated for direct comparison of signature genes (Figure 5C). In H1975 cells, mycoplasma infection affected all six afatinib resistance genes (6/6). Importantly, MYC, E2F1, CCND1, mTOR, AKT1, and PIK3CA are candidate genes implicated in signaling pathways that regulate cell growth and survival.^56^^,^^64^^,^^65^ E2F1 and CCND1 regulate cell cycle progression during the G1/S transition, and mTOR acts as a central nutrient sensor that links growth factor signaling to cellular metabolism.^56^^,^^65^^,^^67^ Additionally, AKT1 and PIK3CA are essential elements of the PI3K/AKT pathway, a major oncogenic signaling axis in cancer.^57^ In PC9 cells, we found 67% overlap in afatinib-resistance genes, namely HIF1A, CA9, LDHA, and BNIP3. This result is consistent with the increased metabolic pathway activity observed in PC9 cells when infected with *Mycoplasma* or becoming resistant to EGFR-TKIs.^68^ Among gefitinib-resistant samples, 33% of genes overlapped (Figure 5C). The overlap in gene signatures and pathway enrichment shows that mycoplasma infection activates molecular programs that parallel those underlying drug resistance.

To validate the overlapping rationale, we performed a directional concordance analysis to show orientation changes (Figure 5D). There was a strong positive correlation between log_2_ fold changes in mycoplasma infection and drug resistance in a scatterplot. Most genes were grouped along the diagonal, indicating that infection and resistance states were regulated similarly. However, about 33% of the genes changed direction, suggesting that both direct pathway mimicry and compensatory regulation contribute to the overall phenotype (Figure 5D). We also analyzed pathway module responses to afatinib in H1975 and PC9 cells (Figure 5E). The diagonal comparison plot showed that the molecules used different strategies. H1975 cells exhibited significant upregulation of genes within the PI3K/AKT/mTOR signaling axis (orange cluster), including RPS6KB1, PIK3CA, mTOR, AKT2, and RPS6KB2. This activation parallels the enrichment of the mTOR signaling pathway noted in both mycoplasma infection and afatinib resistance. Concurrent upregulation of growth/MYC pathway genes (purple cluster) also contributed to the enhanced cell-cycle pathway activity.

### Pathway-level validation demonstrates consistent EGFR pathway activation

**Figure 6:**
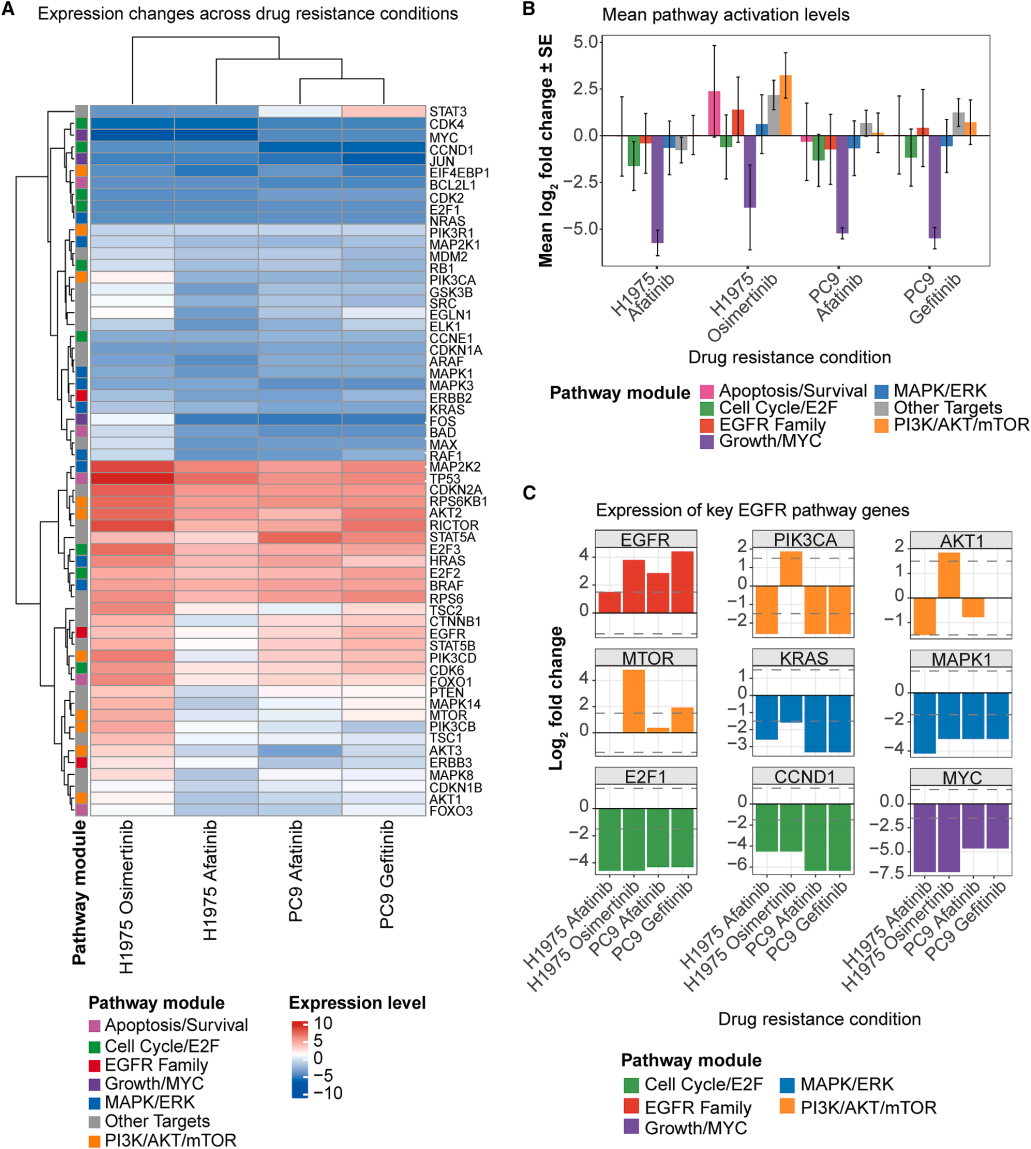
Core EGFR pathway alterations across multiple EGFR-TKI resistance models (A) Heatmap of expression changes for key pathway genes across drug-resistant models (H1975 osimertinib, H1975 afatinib, PC9 afatinib, and PC9 gefitinib). Genes are grouped by functional units. (B) Mean expression shifts for each pathway group across resistance conditions. Bars represent average log_2_ fold change ±SEM (standard error of the mean). (C) Expression profiles of key pathway genes. Visualized representations show cell-line-specific differences at the level of individual genes.

The expression of EGFR pathway genes in the presence of mycoplasma infection and drug resistance remained unclear. Hierarchical clustering revealed distinct expression clusters, indicating how pathways were working together (Figure 6A). The upregulated genes (red cluster) were consistently activated in H1975 osimertinib, H1975 afatinib, PC9 afatinib, and PC9 gefitinib resistance conditions. These genes are directly related to pathways that are more active during mycoplasma infection (mTOR signaling, cell cycle regulation, and PI3K/AKT activation).^56^^,^^57^^,^^67^ Some of the genes that were turned off (in the blue cluster) and cell cycle/E2F genes of CDK4, CCND1, CDK2, E2F1, and E2F2 were mostly upregulated. It has been shown that proliferative signaling is a common way to resist drugs. The average levels of pathway activation across drug-resistance conditions were similar to those observed in pathways under mycoplasma infection (Figure 6B). PI3K/AKT/mTOR signaling (orange bars) was significantly upregulated in H1975 osimertinib and showed moderate increases in other conditions, resembling mTOR signaling pathway enrichment in mycoplasma-infected cells. The significant downregulation in PC9 cells treated with gefitinib indicates that compensatory mechanisms are activated when direct PI3K/AKT activation is inadequate. Cell cycle/E2F modules (green bars) showed bidirectional regulation depending on the situation. This shows the balance between signaling for growth and survival. The mean activation profiles illustrate context-dependent pathway engagement while preserving overall convergence on fundamental resistance mechanisms established during mycoplasma infection. Figure 6C displays the individual gene-expression profiles of key genes in the EGFR pathway: mTOR increased steadily across all H1975 conditions, whereas responses in PC9 varied. This directly supports the hypothesis that enrichment of the mTOR signaling pathway is a common mechanism. Moreover, MYC-associated activity (purple bars) decreased substantially in PC9 conditions but showed only a limited reduction in H1975 conditions. This suggests that PC9 cells depend on MYC-independent survival mechanisms established during mycoplasma infection (Figure 6C).

### Integrated mechanistic model links transcriptomic and functional findings

**Figure 7:**
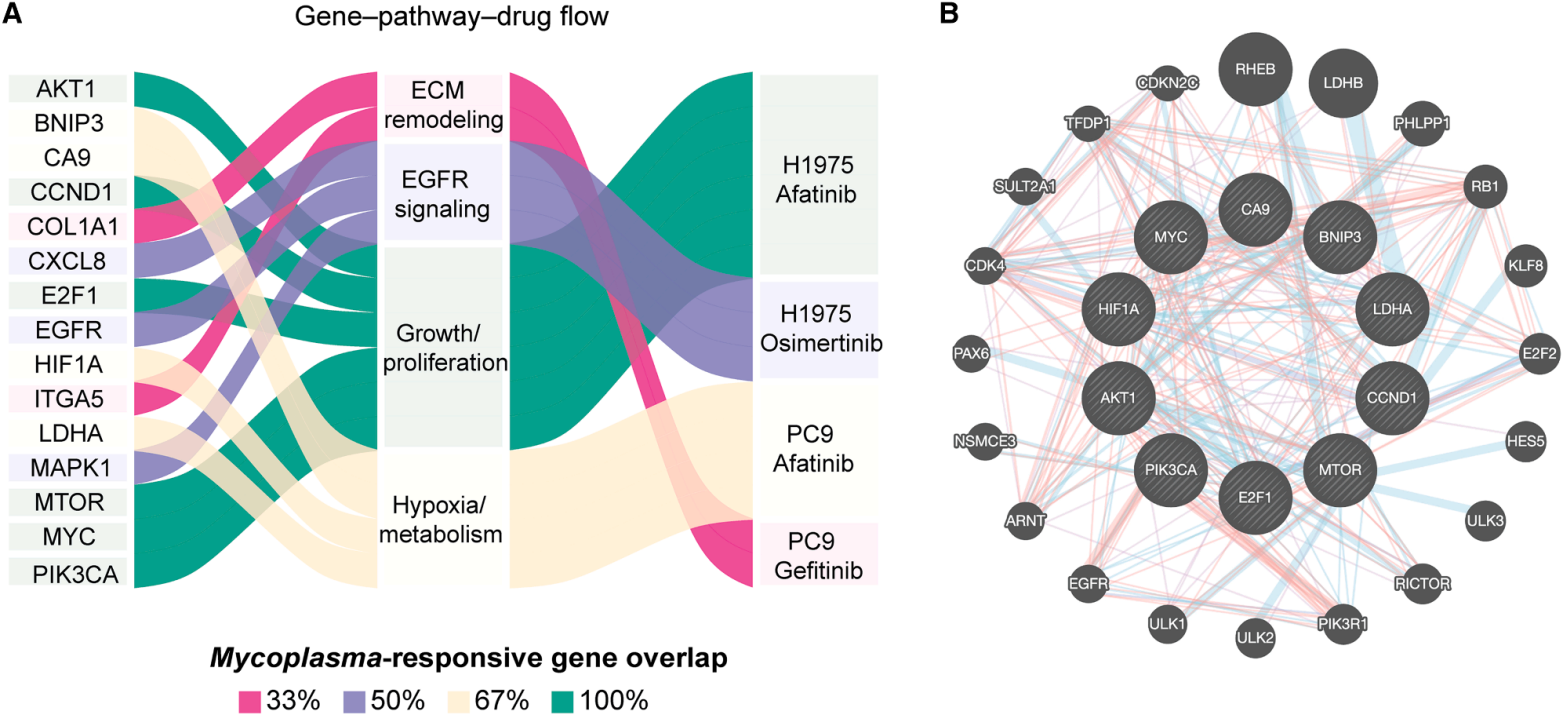
Shared signaling network links mycoplasma-associated response to TKI resistance via gene mapping (A) Sankey diagram illustrates gene-pathway-condition flow relationships. Left: 15 core genes including key drivers. Middle: 4 major signal clusters. Edge width denotes gene contribution strength; color indicates the percentage of pathway genes also responsive to mycoplasma infection. (B) Protein-protein interaction network of resistance genes. Network topology reveals relationships among genes shared across resistance contexts and induced by *Mycoplasma*. Central hub genes highlight the convergence of signaling axes and metabolic reprogramming. Node size denotes degree centrality; edge weights represent interaction confidence.

We summarized the internal flow by a Sankey diagram in Figure 7A. The plot illustrating the distributions of mycoplasma-responsive genes maps into four major resistance pathways—ECM remodeling, EGFR signaling, proliferation, and hypoxia—is depicted in Figure 7A. These transcriptomic data support a unified mechanism whereby *Mycoplasma* activates resistance-linked pathways. Using the GeneMANIA network construction tool, DEGs can be narrowed down into smaller subsets for functional term enrichment and pathway-based analyses. The major regulators, MYC, LDHA, CCND1, mTOR, E2F1, PIK3CA, and AKT1, sit at the crossroads of survival, metabolic adaptation, and proliferation, representing classic drivers of resistance (Figure 7B).^35^^,^^48^^,^^64^^,^^68^ Among these, mTOR and LDHA stand out as central, targetable nodes, connecting mycoplasma-induced changes to EGFR-TKI resistance. Based on their pivotal roles and druggability, we performed functional validation studies targeting mTOR and LDHA with specific drugs, alone and in combination, to evaluate whether their inhibition could reverse resistance phenotypes.

### Machine learning models achieve high predictive accuracy

**Figure 8:**
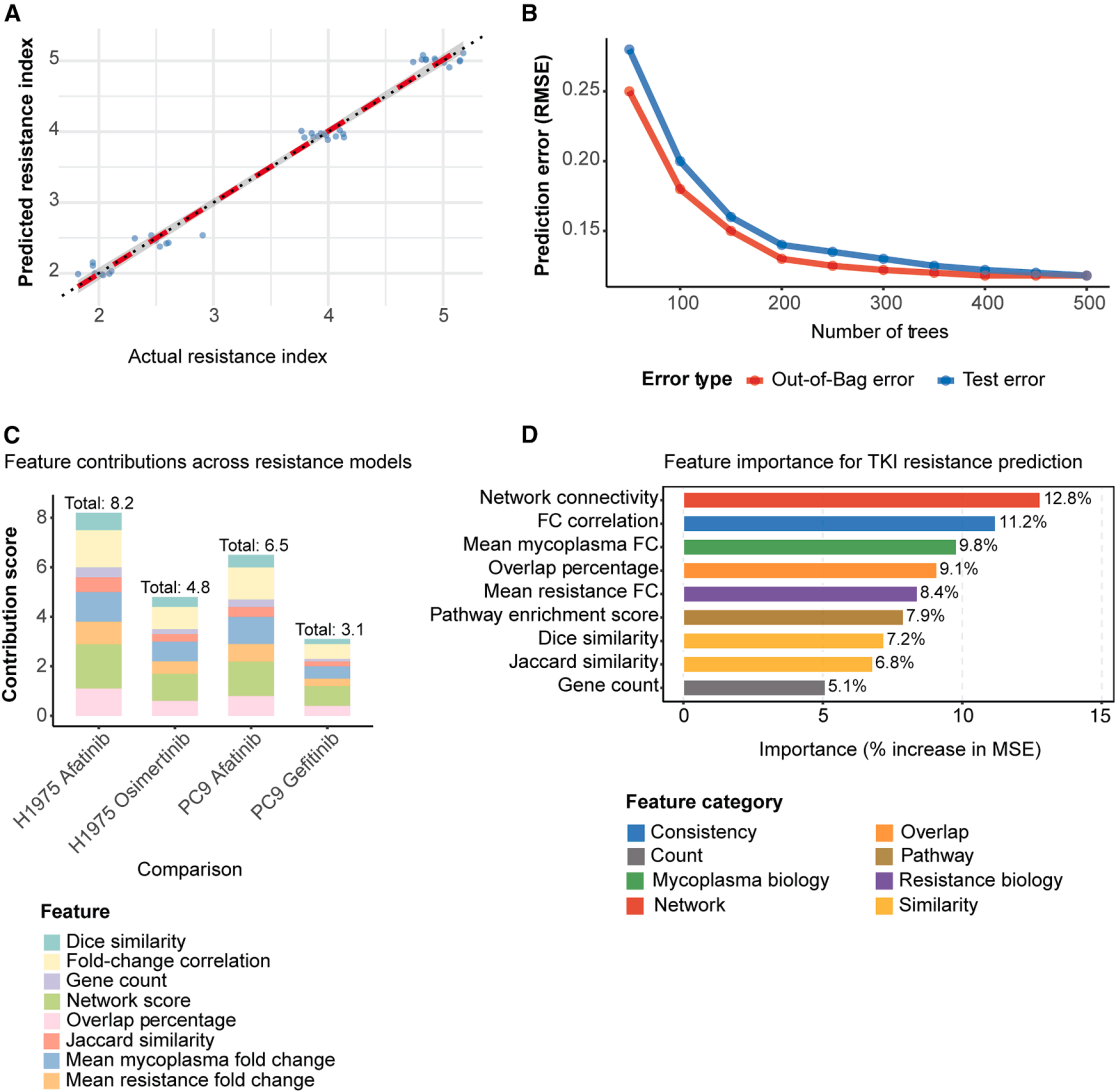
Machine learning identifies convergent features that drive TKI resistance (A) Random forest model performance showing a strong correlation between predicted and observed resistance indices (R^2^ = 0.983; root-mean-square error [RMSE] = 0.149). (B) Model convergence curve tracking prediction error versus number of trees. Both out-of-bag error (red) and test error (blue) converge at approximately 400 trees (RMSE = 0.118), indicating optimal model stability and minimal overfitting. (C) Feature contributions to resistance index predictions across conditions. Stacked bars show the cumulative contribution of 9 features to the total resistance index. (D) Feature importance ranking based on percent increase in mean squared error (MSE).

Random forest classification models combining features from our overlap and pathway analyses (Figure 8) were created to determine whether transcriptional changes caused by *Mycoplasma* can quantitatively predict drug resistance phenotypes. We evaluated the model’s performance by comparing predicted resistance indices with the actual ones (Figure 8A). The model performed well, with an R^2^ of 0.983 and a root-mean-square error (RMSE) of 0.149. The scatterplot revealed strong agreement between the training (red dots) and validation (blue dots) sets, as both clustered closely along the diagonal with limited variation. Model-convergence analysis indicated that an optimal model was achieved with approximately 400 trees (Figure 8B). Both the out-of-bag error (red line) and test error (blue line) declined rapidly from an RMSE of 0.25 at 50 trees to 0.12 at 400 trees, after which they remained constant. This convergence pattern confirms that transcriptional signatures induced by *Mycoplasma* possess authentic predictive information regarding resistance phenotypes (Figure 8B). Figure 8C shows the feature contribution analysis across four drug-cell line comparisons and different participating pathways. H1975 afatinib had the highest total contribution of 8.2, largely due to network connectivity, fold change correlation, and mean mycoplasma fold change. This is consistent with 100% gene overlap and full pathway activation (TNF signaling, IL-17 signaling, mTOR signaling, and endocytosis). H1975 osimertinib had an average contribution with balanced factor inputs, which showed that 50% of the genes were the same. PC9 afatinib had a minor contribution and PC9 gefitinib had the lowest. This is consistent with the reduced gene overlap (33%) and selective pathway activation observed in PC9 cells treated with gefitinib (Figure 8C). Besides, global feature importance analysis found that network connectivity (12.8%) was the most important predictor followed by fold change correlation (11.2%), mean mycoplasma fold change (9.8%), and overlap percentage (9.1%) (Figure 8D). Other important factors were mean resistance fold change, pathway enrichment score, Dice similarity, Jaccard similarity, and gene count. The network topology metrics support the hub-centered resistance architecture identified in our earlier network analysis.

Overall, machine learning validation confirms that mycoplasma-induced transcriptomic signatures, especially changes in pathway enrichment patterns (mTOR signaling, endocytosis, and inflammatory pathways) and network connectivity hold measurable information of drug resistance phenotypes. The elevated predictive accuracy (R^2^ = 0.983) and strong dependence on biological features further support the conclusion that shared pathway activation between mycoplasma infection and drug resistance reflects true mechanistic relationships rather than statistical artifacts.

Altogether, our comprehensive multi-omics analyses elucidate a coherent mechanistic model linking mycoplasma infection to EGFR-TKI resistance through convergent pathway activation. Mycoplasma infection induces cell-line-specific inflammatory responses (TNF, IL-17, and NF-κB signaling) as well as conserved pathway modifications, including mTOR signaling and metabolic reprogramming. The transcriptional changes in infection-driven pathways are similar to those observed in drug-resistant variants.

### Prognostic significance of *Mycoplasma* and LDHA in NSCLC patients treated with EGFR-TKIs

In agreement with previous studies linking mycoplasma infection to worse outcomes in osimertinib-treated patients,^34^ we observed a similar association in our cohort of NSCLC patients. *Mycoplasma* was detected by PCR analysis of RNA isolated from formalin-fixed paraffin-embedded (FFPE) tissues of 64 NSCLC patients (Table S1). We also evaluated LDHA expression, as increased LDHA expression has been associated with a poor prognosis in NSCLC. TCGA (The Cancer Genome Atlas)-GTEx cohort data showed higher LDHA mRNA levels in NSCLC tissues than in normal lung tissues (Figure S6A). LDHA expression was significantly correlated with overall survival (OS) in the survival analysis (Figure S6B). However, it is important to note that the TCGA dataset did not consider EGFR mutations or specific EGFR-TKI therapy, suggesting that LDHA could be a more general prognostic marker in NSCLC regardless of these factors.

To explore the clinical significance of LDHA in the context of EGFR-TKI therapy, we conducted immunohistochemistry (IHC) analysis on the aforementioned tissue samples from the NSCLC patients receiving EGFR-TKIs. Remarkably, 31 out of 35 (88.6%) M+ tissues exhibited high LDHA expression, compared with only 1 out of 29 (3.4%) M- tissues (Figure S6C). Demographic and clinical factors such as sex and smoking history did not correlate with OS. However, survival analysis revealed a significant negative correlation (log rank *p* < 0.001) between LDHA expression and OS in patients treated with EGFR-TKIs (Figure S6D). Representative images of LDHA expression (low and high) are shown in Figure S6E.

### Pharmacological inhibition of mTOR pathway and LDHA reduces cell viability to osimertinib in mycoplasma-resistant cells

To establish causal evidence linking mycoplasma infection to osimertinib resistance, antibiotic eradication experiments were performed in H1975 and PC9 M+ cells. *Mycoplasma* was eradicated through a multi-antibiotic regimen. Mycoplasma-mediated clearance significantly, though partially, restored osimertinib sensitivity at 5, 50, and 100 nM in both cell lines (Figure S7). Partial restoration of osimertinib sensitivity following antibiotic-mediated eradication suggests that *Mycoplasma* contributes to a partially reversible drug-resistant state. The incomplete restoration of sensitivity to mycoplasma-uninfected controls following infection clearance (i.e., 100% eradication of *Mycoplasma*) further suggests that *Mycoplasma* induces a stable transcriptional reprogramming that persists beyond bacterial eradication. This provides a mechanistic rationale for downstream targeted therapy alongside antimicrobial intervention.

The mTOR inhibitor BEZ-235 and the LDHA inhibitor LDHA-IN-8 were evaluated in mycoplasma-resistant H1975 and PC9 cells. Both compounds displayed greater single-agent anti-proliferative activity in M+ EGFR-TKI-resistant cells compared with parental cells (Figure S8A), consistent with the upregulation of their respective targets. However, neither inhibitor alone was sufficient to fully overcome the EGFR-TKI resistance. To enhance pathway inhibition, BEZ-235 or LDHA-IN-8 was combined with osimertinib at their respective 25% growth inhibition concentrations (GI25). After 72 h, BEZ-235 plus osimertinib or LDHA-IN-8 plus osimertinib reduced cell viability by approximately 50%, and 60%, respectively, in resistant cells (Figure S8B). Notably, the triple combination of BEZ-235, LDHA-IN-8, and osimertinib achieved the strongest anti-proliferative effect, reducing cell viability by ∼80%, indicating a markedly enhanced effect of concurrently targeting mTOR, LDHA, and EGFR to counteract mycoplasma-driven resistance. We extended the combination experiments to M+ PC9 cells to assess whether these findings generalize across EGFR-mutant backgrounds (Figure S8C). Single-agent osimertinib, BEZ-235, and LDHA-IN-8 each induced slight to moderate growth inhibition in M+ PC9 cells, which is consistent with H1975 results. Dual combination of osimertinib with either BEZ-235 or LDHA-IN-8 reduced relative cell growth to approximately 45%–50%; meanwhile, the triple combination of osimertinib, BEZ-235, and LDHA-IN-8 reduced relative cell growth to nearly 10% in M+ PC9 cells. These findings represent a more pronounced suppression than observed in H1975 M+ cells (around 20%) and confirm potent synergistic efficacy of concurrent mTOR, LDHA, and EGFR inhibition over two independent EGFR-mutant backgrounds.

## Discussion

In this study, we performed transcriptomic analysis, functional studies, and pharmacological and clinical validation analyses to explore the intricate relationship between mycoplasma infection and EGFR-TKI resistance. Interestingly, our findings reveal significant molecular convergence: mycoplasma infection activates pathways that mediate acquired resistance to various TKIs.

Functional studies confirmed transcriptomic predictions. Cells infected with *Mycoplasma* showed a significantly lower level of EGFR internalization (5%–10% compared with 20% in controls and *p* < 0.05), confirming that the endocytosis pathway was enriched in all resistance conditions. Also, impaired internalization was associated with increased receptor phosphorylation and ongoing downstream signaling (phospho-AKT *p* < 0.01 and phospho-ERK *p* < 0.001). The above validation proves two complementary mechanisms: disrupted EGFR trafficking blocks receptor downregulation and surface availability, and persistent phosphorylation sustains PI3K/AKT/mTOR signaling under EGFR-TKI exposure.

Mechanistically, M+ cells exhibited defective EGFR internalization, which correlated with chronic activation of downstream pathways. Consistent with transcriptomic profiling, we observed a downregulation of signatures related to the endocytic pathway, including genes involved in receptor trafficking such as CLTC, CBL, and RAB family members.^36^ The downregulation of clathrin expression was also observed at the protein level, using a specific ELISA. Together, these data indicate that dysregulated receptor trafficking may be a contributing factor to sustained EGFR signaling.

M+ cells exhibited increased NF-κB activation together with enrichment of inflammatory signaling pathways, including TNF- and IL-17-related programs.^46^ Although H1975 and PC9 cells displayed partially distinct transcriptomic signatures, both models converged on common resistance-associated pathways, particularly mTOR activation, inflammatory signaling, and metabolic reprogramming. The stronger NF-κB-related enrichment observed in H1975 cells may reflect differences in the underlying oncogenic signaling context between the two EGFR-mutant models. However, LPS exposure alone failed to induce the resistance phenotype, suggesting that generalized inflammatory activation does not fully account for the observed effects.

Transcriptomic analysis demonstrated cell-line-specific inflammatory responses. H1975 cells primarily activated TNF, IL-17, and NF-κB signaling, and PC9 cells additionally engaged ECM remodeling and focal adhesion pathways. These pathways closely capture the characteristics of drug-resistant cells,^50^^,^^69^ suggesting that mycoplasma infection primes cells for resistance through a coordinated network-level mechanism rather than single molecular variation. Furthermore, we discovered substantial molecular convergence at the transcriptional level: mycoplasma infection activates the same pathways and genes that promote acquired drug resistance across diverse TKIs. The percentage of gene overlap ranged from 33% to 100%, depending on the cell line and drug context. For H1975 afatinib resistance, there was 100% agreement. Machine learning models achieved exceptional predictive accuracy and validated the assumption that mycoplasma-induced transcriptional signatures quantitatively predict resistance phenotypes. These outcomes support our hypothesis that mycoplasma infection operates as a “priming” resistance mechanism via convergent pathway activation rather than discrete molecular alterations.

Network analysis revealed that various alterations induced by *Mycoplasma* converge at several highly interconnected regulatory hubs. These key genes integrate signals from inflammatory pathways and metabolic reprogramming (mTOR and LDHA) with changes in trafficking, including endocytosis and phosphatidylinositol signaling. The hub-based architecture elucidates the conservation of resistance mechanisms across various drugs: mycoplasma infection activates multiple pathway modules simultaneously, as suggested in previous studies.^53^ These modules can be activated via different mechanisms, depending on the TKI and the cell type. However, in our multi-method validation (pathway analysis, overlap analysis, and network analysis) all converge on the same biological conclusions.

Furthermore, subsequent validation studies with specific mTOR and LDHA inhibitors demonstrated the potential to effectively reverse mycoplasma-associated EGFR-TKI resistance. This implies that metabolic and signaling rewiring induced by *Mycoplasma* creates vulnerabilities that can be therapeutically exploited. Combination of EGFR-TKIs and inhibitors of these key downstream nodes could be a promising strategy to overcome or prevent resistance.

Notably, antibiotic-mediated rescue further distinguished the effects of pathogen clearance from those of pathway inhibition in overcoming resistance associated with mycoplasma infection. While antibiotic-induced clearance partially reversed osimertinib resistance, an incomplete rescue of the phenotype indicates the presence of infection-induced transcriptional changes. We observed pronounced growth suppression when combining mTOR and LDHA inhibition with osimertinib in mycoplasma-infected cells. Accordingly, this observation leads to a complementary therapeutic framework: antibiotic treatment targets the upstream infectious driver, and downstream resistance mechanisms are targeted through pathway-based therapy. Such a strategy may have implications for future clinical approaches incorporating mycoplasma screening and combination therapy in EGFR-mutant NSCLC.

However, while LDHA inhibition represents a potential therapeutic strategy, systemic metabolic toxicity remains a consideration, as glycolytic pathways are also essential in normal tissues. LDHA is upregulated in tumor cells as part of the Warburg-associated glycolytic phenotype, where it supports NAD+ regeneration and sustained glycolytic flux, while its expression is more restricted in most normal adult tissues, therefore providing a degree of tumor selectivity.^54^ Preclinical data on LDHA inhibitors demonstrate selective antiproliferative activity in LDHA-dependent cancer models with acceptable tolerability in normal cell systems.^70^ Importantly, human genetic evidence supports metabolic redundancy under basal physiological conditions: individuals with inherited LDHA deficiency (glycogen storage disease type XI) survive to adulthood and present primarily with exercise-induced symptoms, including exertional myalgia and occasional rhabdomyolysis during intense anaerobic activity but maintain normal physiological function at rest.^71^ This suggests that systemic LDHA inhibition may be tolerated under non-strenuous conditions, though context-specific vulnerability in hypoxic tissues requires careful evaluation.

Mycoplasma colonization is common among lung cancer patients, and its asymptomatic nature makes it an overlooked contributor to TKI resistance.^33^ There are studies that indicate a high prevalence, with one study finding that 52.6% of lung cancer cases are positive for *Mycoplasma* species,^33^^,^^72^ while another study observed *M. hyorhinis* in 60.4% of lung adenocarcinoma patients.^34^ We observed a similar percentage in our cohort. In addition, both mycoplasma infection and elevated LDHA levels detected in M+ tissues correlated with shorter survival, suggesting that they may serve as prognostic markers for NSCLC patients undergoing EGFR-TKI therapy.

These findings align with the growing evidence that the intratumoral microbiome constitutes a functionally relevant component of the tumor microenvironment. Prior landmark studies have indicated that intratumoral bacteria can inactivate chemotherapeutic agents enzymatically, and gut microbiome composition modulates responses to immune checkpoint inhibitors.^30^^,^^73^^,^^74^ Here, we extend this paradigm to targeted therapy by showing that persistent mycoplasma infection contributes to EGFR-TKI resistance in EGFR-mutant NSCLC. Unlike somatic mutations, infection-driven resistance may be at least relatively reversible through antimicrobial intervention. Prospective evaluation of mycoplasma screening and eradication as an adjunct to EGFR-TKI therapy represents a translational priority arising from this work.

In summary, our study supports a model, in which mycoplasma infection promotes resistance-associated signaling and metabolic reprogramming in EGFR-mutant NSCLC. Integrated transcriptomic, functional, and clinical analyses identify mycoplasma infection as a causal, non-genetic contributor to EGFR-TKI resistance and highlight convergent pathways, including mTOR signaling, glycolytic metabolism, and impaired receptor trafficking, as actionable therapeutic vulnerabilities. Although this study used a simple machine learning model to predict, predictor significance in a small sample, the strategies and methodologies can be applied in practice under the same conditions, thereby paving the way for new biomarker discoveries, with the potential to improve NSCLC treatment and outcomes.

### Limitations of the study

Several limitations should be considered in our study. First, our analyses were conducted by using two well-characterized NSCLC cell lines, but broader validation in additional models, including patient-derived samples or organoids, will be needed to confirm the generalizability and clinical relevance of our findings. Similarly, the cohort of patients was small, but we were able to confirm previous findings on the prognostic role of both mycoplasma infection and LDHA expression.^34^^,^^75^

Second, although we confirmed the presence of *Mycoplasma*, the characterization of *Mycoplasma* species was limited, and different species may modulate EGFR signaling and drug responsiveness in distinct ways.

Pharmacological inhibition provided consistent functional evidence supporting LDHA and mTOR as potential therapeutic vulnerabilities; however, genetic silencing approaches were not performed in the present study. Therefore, additional mechanistic validation using genetic approaches will be important to further confirm the specific contribution of these pathways to mycoplasma-associated resistance.

Although antibiotic-mediated mycoplasma clearance was incomplete, the partial restoration of osimertinib sensitivity indicates the resistance phenotype is reversible. Reinfection experiments and long-term recovery studies were not performed in this work but leave the possibility to further define the reversibility and stability of the resistance phenotype. However, the potential clinical benefit of antimicrobial therapy in EGFR-TKI-resistant NSCLC remains to be determined.

The internal clinical cohort included 64 patients, providing a strong foundation for evaluating mycoplasma prevalence and its association with clinical outcomes. However, prospective validation in larger cohorts will be required to establish clinical utility and to evaluate the feasibility of microbial screening strategies.

Future directions should include both (1) the inclusion of more cell line panels and other preclinical models such as patient-derived cells/organoid models and (2) clinical studies in larger cohorts examining the correlation between mycoplasma status and TKI response in NSCLC patients. In these studies, the machine learning framework could be applied to predict resistance in clinical samples by using transcriptomic sketches. To this goal, a neural network would be considered an optimal tool for visualizing and detecting predictive patterns.^76^

## Resource availability

### Lead contact

Request for further information and resources should be directed to and will be fulfilled by the lead contact, Elisa Giovannetti (e.giovannetti@amsterdamumc.nl).

### Materials availability

This study did not generate new unique reagents. Drug-resistant cell line derivatives generated in this study are available from the lead contact upon reasonable request.

### Data and code availability

• RNA sequencing data have been deposited at the NCBI Gene Expression Omnibus (GEO) database under accession number GSE314455.
• All original code has been deposited at GitHub and is publicly available at https://github.com/bingwang1/nsclc.
• Any additional information required to reanalyze the data reported in this paper is available from the lead contact upon request.

## Acknowledgments

The authors thank Paolo Aretini and Myriam Capula (FPS) for their technical support with sequencing. The authors also thank Ida van de Meulen-Muileman and Monique van Eijndhoven (Amsterdam UMC) for their technical assistance and valuable discussions related to KWF grant nos. 13598 and 15305.

This study was supported by research grants from the Dutch Cancer Society (to E.G. and D.D.). The authors also acknowledge financial support from the 10.13039/501100004543China Scholarship Council (CSC) for the PhD fellowship of B.W. (202407720066).

## Author contributions

Conceptualization, B.W., V.D., A.G., and E.G.; methodology, B.W., V.D., A.G., A.L., A.P., and E.G.; investigation, B.W., V.D., A.G., A.L., A.P., and E.G.; writing – original draft, B.W. and E.G.; writing – review & editing, B.W. and E.G.; funding acquisition, E.G., D.D., and B.W.; resources E.G. and D.D.; supervision, E.G.

## Declaration of interests

The authors declare no competing interests.

## STAR★Methods

### Key resources table

**Table:** 

REAGENT or RESOURCE	SOURCE	IDENTIFIER
**Antibodies**		

Rabbit monoclonal anti-phospho-EGFR (Y1068)	Cell Signaling Technology	Cat#3777S; RRID: AB_2096270
Rabbit monoclonal anti-EEA1	Cell Signaling Technology	Cat#2411S; RRID: AB_2096814
TRITC-conjugated anti-LAMP3	Santa Cruz Biotechnology	Cat#sc-5275; RRID: AB_627877
Alexa Fluor 488-conjugated anti-rabbit secondary antibody	Abcam	Cat#ab150077; RRID: AB_2576217
Mouse monoclonal anti-EGFR	Invitrogen	Cat#MA5-13319; RRID: AB_10985841
Anti-mouse HRP-conjugated secondary antibody	Cell Signaling Technology	Cat#7076S; RRID: AB_330924
Rabbit polyclonal anti-LDH-A	Abcam	Cat#ab9002; RRID: AB_306721

**Biological samples**		

Human FFPE NSCLC specimens	Amsterdam UMC/VU Medical Center, Parma University Hospital, Antwerp University Hospital	Ethics approvals: BUP2016-032; 18379; B300201316249

**Chemicals, peptides, and recombinant proteins**		

Osimertinib	Selleck Chemicals	Cat#S7297
Afatinib	Selleck Chemicals	Cat#S1011
Gefitinib	Selleck Chemicals	Cat#S1025
Epidermal growth Factor (EGF)	Thermo Fisher Scientific	Cat#PHG0311
Ciprofloxacin	Sigma-Aldrich	Cat#17850
BM-Cyclin 1 (tiamulin derivative)	Roche Diagnostics	N/A
BM-Cyclin 2 (minocycline derivative)	Roche Diagnostics	N/A
TRIzol reagent	Thermo Fisher Scientific	Cat#15596018
Lipopolysaccharide (LPS)	Sigma-Aldrich	Cat#L2630
Paraformaldehyde	Sigma-Aldrich	Cat#P6148
Triton X-100	Sigma-Aldrich	Cat#T8787
DAPI	GERBU Biotechnik GmbH	Cat#1050-0025
Lowry reagent	Sigma-Aldrich	N/A

**Critical commercial assays**		

MycoAlert Mycoplasma Detection Kit	Westburg	Cat#LT07-218
BM-Cyclin Mycoplasma Elimination Kit	Roche Diagnostics	Cat#10799050001
Ambion RecoverAll Total Nucleic Acid Isolation Kit	Life technologies	N/A
TaqMan Universal Master Kit	Thermo Fisher Scientific	Cat#4304437
First Strand cDNA Synthesis Kit	Thermo Fisher Scientific	Cat#K1612
PathScan Phospho-NF-κB p65 (Ser536) Sandwich ELISA Kit	Cell Signaling Technology	Cat#7173
Human Clathrin ELISA Kit	Meimian	Cat#MM-51085H1

**Deposited data**		

RNA-seq raw and processed data	NCBI Gene Expression Omnibus	GEO: GSE314455
Transcriptomic analysis code	GitHub	https://github.com/bingwang1/nsclc

**Experimental models: Cell lines**		

Human: H1975 NSCLC cell line (EGFR L858R + T790M)	ATCC	RRID: CVCL_1511
Human: PC9 NSCLC cell line (EGFR exon 19 deletion)	ATCC	RRID: CVCL_B260

**Software and algorithms**		

R (v4.3.0)	R Foundation for Statistical Computing	http://www.r-project.org/; RRID: SCR_001905
GraphPad Prism (v9.5.1)	GraphPad Software	http://www.graphpad.com/; RRID: SCR_002798
ImageJ	NIH	https://imagej.net/; RRID: SCR_003070
FastQC (v0.11.9)	Babraham Bioinformatics	http://www.bioinformatics.babraham.ac.uk/projects/fastqc/; RRID: SCR_014583
STAR (v2.7.3a)	Dobin et al.^37^	https://github.com/alexdobin/STAR; N/A
DESeq2 (v1.40.2)	Bioconductor	https://bioconductor.org/packages/release/bioc/html/DESeq2.html; RRID: SCR_015687
featureCounts (v2.0.1)	Subread project	https://subread.sourceforge.net/
GeneMANIA (v3.6.0)	Warde-Farley et al.^77^	http://genemania.org/; RRID: SCR_005709

### Experimental model and study participant details

#### Cell lines

H1975 (L858R+T790M mutations) and PC9 (exon 19 deletion) cell lines were obtained from American Type Culture Collection (ATCC, Manassas, VA, USA), and maintained in RPMI medium supplemented with 10% FBS, 1% penicillin/streptomycin, and 2mM HEPES at 37 °C in 5% CO_2_. The NSCLC cell lines used in this study had previously been characterized for *EGFR* and *KRAS* mutations and were tested for their authentication by PCR profiling at BaseClear (Leiden, The Netherlands).

#### Human participants and clinical samples

Sixty-four NSCLC patients treated with first-generation EGFR-TKIs and/or osimertinib, including 43 patients with advanced EGFR-mutated NSCLC who were both TKI-naïve or in progression after first-line therapy with EGFR-TKI for the onset of T790M mutation were included. The studies on these clinical samples were approved by the local Ethics Committees of Amsterdam UMC/VU Medical Center (Amsterdam, the Netherlands) biobank (protocol number BUP2016-032), in agreement with the “Code for Proper Secondary Use of Human Tissue in the Netherlands” developed by the Dutch Federation of Medical Scientific Societies (FMWV), Parma University Hospital (protocol number 18379) and Antwerp University Hospital (protocol number B300201316249). Patient characteristics at baseline are summarized in Table S1.

### Method details

#### Mycoplasma detection and species identification

Mycoplasma-positive and mycoplasma-negative cultures were handled separately with appropriate sterilization procedures. Mycoplasma contamination was routinely assessed using the MycoAlert Mycoplasma Detection Kit (Westburg, Leusden, The Netherlands) following the manufacturer’s protocol. To determine the specific *Mycoplasma* species present, including the detection of *M. hyorhinis*, additional PCR-based analyses were performed, as reported by Tocqueville et al.^78^ This approach employs species-specific primer sets targeting conserved *Mycoplasma* genomic regions, enabling discrimination between different contaminating *Mycoplasma* species commonly detected in cell culture systems. These analyses consistently confirmed the presence of *M. hyorhinis* throughout the experimental procedures. While these methods allowed robust detection and species identification, a systematic quantitative assessment of mycoplasma burden was not performed; however, semi-quantitative analyses suggested comparable infection levels across experimental conditions.

#### Mycoplasma clearance and antibiotic rescue assays

The antibiotic rescue was performed using a multi-antibiotic strategy, including both ciprofloxacin (20 μg/mL; Sigma-Aldrich, St. Louis, MO, USA) and BM-Cyclin (Roche Diagnostics), including BM-Cyclin 1 (tiamulin derivative, 10 μg/mL) and BM-Cyclin 2 (minocycline derivative, 5 μg/mL) according to the manufacturer’s protocol. Cells were routinely tested for mycoplasma infection at each passage. This strategy resulted in 100% eradication rates after 2 weeks of treatment. Following clearance, cells were treated with osimertinib to evaluate the restoration of drug sensitivity.

#### Establishment and genetic characterization of EGFR-TKI-resistant cells

*In vitro* acquired resistance to osimertinib, afatinib, or gefitinib was modeled by applying dose-escalation (up to 1 μM) of these compounds to H1975 and PC9 cells. Genomic DNA was extracted using the Ambion®-RecoverAll kit (Life Technologies, Breda, The Netherlands), and the mutational status of *EGFR*, *KRAS*, *BRAF*, and *PIK3CA* was determined as previously described.^79^ We used quantitative RT-PCR to measure *EGFR* mRNA expression levels, potentially linked to gene amplification or increased transcription. RNA was extracted using TRIzol (Thermo Fisher Scientific, #15596018), and cDNA synthesis was performed with the First Strand cDNA Synthesis Kit (Thermo Fisher Scientific, #K1612). *EGFR* and *β-actin* expression were quantified with TaqMan Universal Master Mix, and relative expression was calculated using the 2-ΔΔCT method.

#### Cell viability assay

Cell viability was measured using the Sulforhodamine B (SRB) assay. Cells (3500 per well) were seeded in 96-well plates. To ensure experimental growth, each cell line was seeded at a different density depending on its growth rate. To optimize the seeding density, a growth curve for each unknown cell line was recorded prior to the SRB experiment. After 24 hours, cells were treated with serial concentrations of afatinib or osimertinib for 72 hours. Cultures were fixed in 10% trichloroacetic acid for ≥1 hour at 4°C, washed five times with running water and air-dried prior to SRB staining.

Additional SRB studies with mTOR and the LDHA inhibitors were performed to assess whether the specific inhibition of common emerging resistance pathways could reduce the cell proliferation in mycoplasma-resistant cells.

#### Morphological evaluation and immunofluorescence staining

To assess potential morphological and cellular changes induced by mycoplasma infection, we performed both a standard morphological evaluation and an immunofluorescence analysis comparing mycoplasma-infected and non-infected cells.

H1975 cells (25000 cells/100 μL) were seeded on 18 × 18mm coverslips (Menzel Glaser) and cultured for 24 hours. Cells were fixed with 4% paraformaldehyde for 10 minutes, washed and permeabilized with 0.05% Triton X-100 in PBS for 30 minutes. After blocking with 10% BSA for 1 hour at room temperature, cells were incubated overnight with primary antibody, (p-EGFR Y1068, Cell Signaling Technologies #3777S 1:250; LAMP-3 TRITC-conjugated, Santa Cruz Biotechnology, #sc-5275; EEA1, Cell signaling Technologies #2411S, 1:150) in BSA 10% and incubated overnight at 4 °C, followed by Alexa Fluor 488-conjugated anti-rabbit secondary antibody (Abcam, #AB150077; dilution 1:1000) for 1 hour at room temperature in the dark. Nuclei were stained with DAPI (GERBU Biotechnik GmbH, #1050-0025; dilution 1:5000). Images were acquired under fluorescent microscope using a 63X oil-immersion objective. Pearson’s correlation coefficients were calculated in ImageJ/Fiji following manual background subtraction.

#### EGFR internalization assay

H1975 cells were seeded on collagen-coated 96-well plates (25 μg/mL). After 24 hours of serum starvation, cells were treated with osimertinib (10nM) or EGF (100 ng/mL) for 1 hour. Cells were washed, fixed with 4% paraformaldehyde for 10 minutes and processed with or without permeabilization (0.05% Triton X-100, 20 minutes) to detect total and surface EGFR. All cells were blocked with 5% BSA for 1 hour, then incubated with an EGFR primary antibody (Invitrogen, #MA5-13319; 1:1000). Next, cells were incubated with 100 μL of secondary antibody (anti mouse HRP-conjugated, Cell Signaling Technologies, #7076S; diluted 1:1000 in 5% BSA) for 1 hour and with 100 uL TMB solution (R&D System, #DY999). The reaction was then stopped with 50 uL of H2SO4 1.0 M and the OD was read at 450 nM. Internalization percentage was calculated as (Total EGFR–Surface EGFR)/Total EGFR ⋅ 100.

#### Phosphoprotein enzyme-linked immunosorbent assay (ELISA) for downstream pathways

Protein lysates were prepared for ELISA and analyzed in triplicate. H1975 and PC9 cells (80% confluence) were treated with osimertinib (10 nM and 1 μM) or vehicle for 1 hour, then stimulated with EGF (100ng/mL, 15mins). Cells were lysed in whole-cell lysis buffer (Cell Signaling Technology, #9803) containing protease/phosphatase inhibitors. Total and phosphorylated AKT (S473) and ERK (T202/Y204) were quantified using ELISA Kits (Biosource International, Camarillo, CA). After overnight incubation at 4 °C, the solution was removed from the wells, and 100 μL of rabbit anti-phospho or rabbit anti–full-length antibodies were added to the corresponding wells of the phospho and full-length AKT and ERK ELISAs. Plates were incubated for 1 hour at room temperature, washed four times, and then incubated with 100 μL of horseradish peroxidase–conjugated anti-rabbit IgG. After 30 minutes, the wells were emptied and a chromogenic substrate solution was added. Following a further 30 minutes, the reactions were stopped with 100 μL of stop solution, and absorbance was measured at 450 nm after 20 minutes. Phospho- and full-length AKT and ERK concentrations were calculated using a standard-curve method, and values were normalized to total protein content determined with the Lowry reagent (Sigma-Aldrich).

NF-κB pathway activation was assessed using the PathScan®Phospho-NF-κB p65 (Ser536) Sandwich ELISA Kit #7173 (Cell Signaling Technology, Danvers, MA, USA) according to the manufacturer’s instructions. Cell lysates were prepared under non-denaturing conditions, and phospho-p65 levels were normalized to total protein concentration. Results were expressed relative to non-infected control cells.

Clathrin levels were quantified using a Human Clathrin ELISA Kit (#MM-51085H1, Meimian, China), according to the manufacturer’s instructions.^69^ Briefly, H1975 and PC9 cell pellets were harvested and lysed under standard lysis conditions. Cell lysates were then added to the appropriate ELISA plates, and after incubation and washing as per the protocols, absorbance was measured using the BioTek Synergy HT plate reader (BioTek Instruments Inc.). Clathrin concentrations were normalized to total protein content to correct for variation in cell number and lysate input.^69^ Normalized values were expressed as a percentage of the control group (non-infected cells).

As a control for generalized inflammatory activation, mycoplasma-negative cells were treated with lipopolysaccharide (LPS; 0.1–10 μg/mL) before osimertinib treatment.

#### RNA sequencing and transcriptomic analysis

Total RNA was isolated using TRIzol (Thermo Fisher Scientific, #15596018). Libraries were prepared using standard protocols and sequenced on Illumina platforms. Raw reads were assessed with FastQC (v0.11.9). Reads were aligned to the human reference genome (GRCh38/hg38) using STAR (v2.7.3a), and uniquely aligned reads were counted using featureCounts (Subread v2.0.1) with GENCODE release 44 annotations. Gene counts were normalized using the median-of-ratios method in DESeq2. Standard quality control procedures confirmed high-quality sequencing data across all samples.

#### Differential expression analysis

DESeq2 (v1.40.2) was applied to identify differentially expressed genes (DEGs) with a negative binomial generalized linear model and Benjamini-Hochberg FDR correction. DEGs were defined as |log_2_ fold change| > 1 and adjusted *p* < 0.05. Fold-change shrinkage was applied to improve estimates for low-count genes.

#### Pathway enrichment and GSEA

We used the Gene Ontology (GO), Kyoto Encyclopedia of Genes and Genomes (KEGG), and Reactome databases and the Hallmark gene sets to conduct functional enrichment analyses. Significant pathways were defined as having a false discovery rate (FDR)-corrected q-value ≤ 0.05 (Benjamini-Hochberg method) with a minimum overlap of 3 genes. Pathways meeting these criteria across multiple independent databases were considered robust. Gene Set Enrichment Analysis (GSEA) was performed using pre-ranked gene lists to compare biological states between mycoplasma-infected and drug-resistant samples. Pathways were ranked by normalized enrichment score (NES). Directional concordance was also evaluated to test whether infection-induced upregulation or downregulation recapitulated resistance-associated changes.

To investigate the role of endocytic regulators, we performed GSEA using GO Biological Process pathways to identify molecular changes associated with impaired EGFR internalization. The leading contributors to pathway enrichment were identified and used for biological interpretation.

#### Overlap analysis

We assessed the intersection of DEGs from infection and resistance datasets using hypergeometric testing and fold enrichment metrics. Overlap coefficients were computed to quantify the degree of concordance between signatures.

#### Network analysis

GeneMANIA (version 3.6.0, https://genemania.org/) was used to generate gene-gene interaction networks and visualize functional relationships among overlapping gene sets.^77^ Network edges were weighted by interaction confidence scores derived from co-expression, pathway co-occurrence and interactions data.

#### Machine learning modeling

Random forest regression models were trained to predict drug resistance phenotypes from mycoplasma-induced transcriptional features. Models were validated using bootstrap resampling. We assessed model performance by the coefficient of determination (R^2^) between predicted and observed resistance indices. Feature importance scores were calculated to identify key predictive genes.

#### Immunohistochemistry

Immunohistochemistry (IHC) to assess LDHA expression was performed using the anti-LDH-A (dilution 1:100; ab9002, Abcam) antibody. The slides were deparaffinized using xylene, rehydrated in alcohol and microwaved at 400W (2 times, for 5 minutes). Immunostaining was performed by the avidin-biotin peroxidase complex technique. Samples were classified as “high” or “low” LDHA based on a previously proposed grading system: strong cytoplasmic expression in >50% of cancer cells or nuclear expression in >10% of cancer cells were defined as high expression.^70^

### Quantification and statistical analysis

#### Study design and data structure

We used two complementary transcriptomic datasets to examine the interaction between mycoplasma infection and drug resistance in NSCLC. The first dataset included transcriptomic profiles of H1975 and PC9 cell lines cultured under mycoplasma-positive or mycoplasma-negative conditions, enabling characterization of infection-driven transcriptional changes. The second dataset comprised transcriptomes from drug-resistant derivatives: specifically, H1975 cells with acquired afatinib or osimertinib resistance, and PC9 cells resistant to afatinib or gefitinib. These samples captured molecular alterations of drug-selected resistance. Through dual-dataset integration, we aimed to distinguish infection-specific effects from resistance-associated alterations and to identify convergent pathways that may mediate mycoplasma-associated therapeutic resistance.

#### Analytical framework

Figure 1 outlines an overview of the computational workflow used in this study. Two RNA-seq datasets were processed: mycoplasma infection profile (Dataset 1) and drug-resistance signatures (Dataset 2). The unified pipeline incorporates standardized preprocessing and quality control, normalization, and differential expression analysis to identify DEGs under infection and resistance conditions. Cross-dataset integration included: (1) gene-level overlap analysis, (2) directional concordance assessment, (3) network topology features, and (4) machine learning-based predictive modeling using random forest regression.

#### Statistical analysis

We used DESeq2 (v1.40.2) for RNA-seq analyses.^38^ IC_50_ values were determined in GraphPad Prism (v9.5.1) using nonlinear regression. Paired tests were applied to compare normalized gene expression. Most data represent ≥3 independent experiments performed in triplicate and are reported as mean ± SEM. We applied two-way ANOVA followed by Tukey’s test for group comparisons. A significance of *p* < 0.05 was considered statistically significant. All statistical analyses were performed in R (v4.3.0).
