# Supplementary material for: Mycoplasma infection drives EGFR-TKI resistance through convergent transcriptomic reprogramming in non-small cell lung cancer
Source: iScience. 2026 Jul 31;29(8):116999. doi: 10.1016/j.isci.2026.116999 (PMC13453009; doi:10.1016/j.isci.2026.116999)
Supplement: Supplementary file 1 [file mmc1.pdf]

iScience, Volume *29*

Supplemental information

Mycoplasma infection drives EGFR-TKI resistance through convergent transcriptomic reprogramming in non-small cell lung cancer

Bing Wang, Valentina Donati, Alessandro Gregori, Alessandro Leonetti, Aldo Pastore, Dongmei Deng, and Elisa Giovannetti

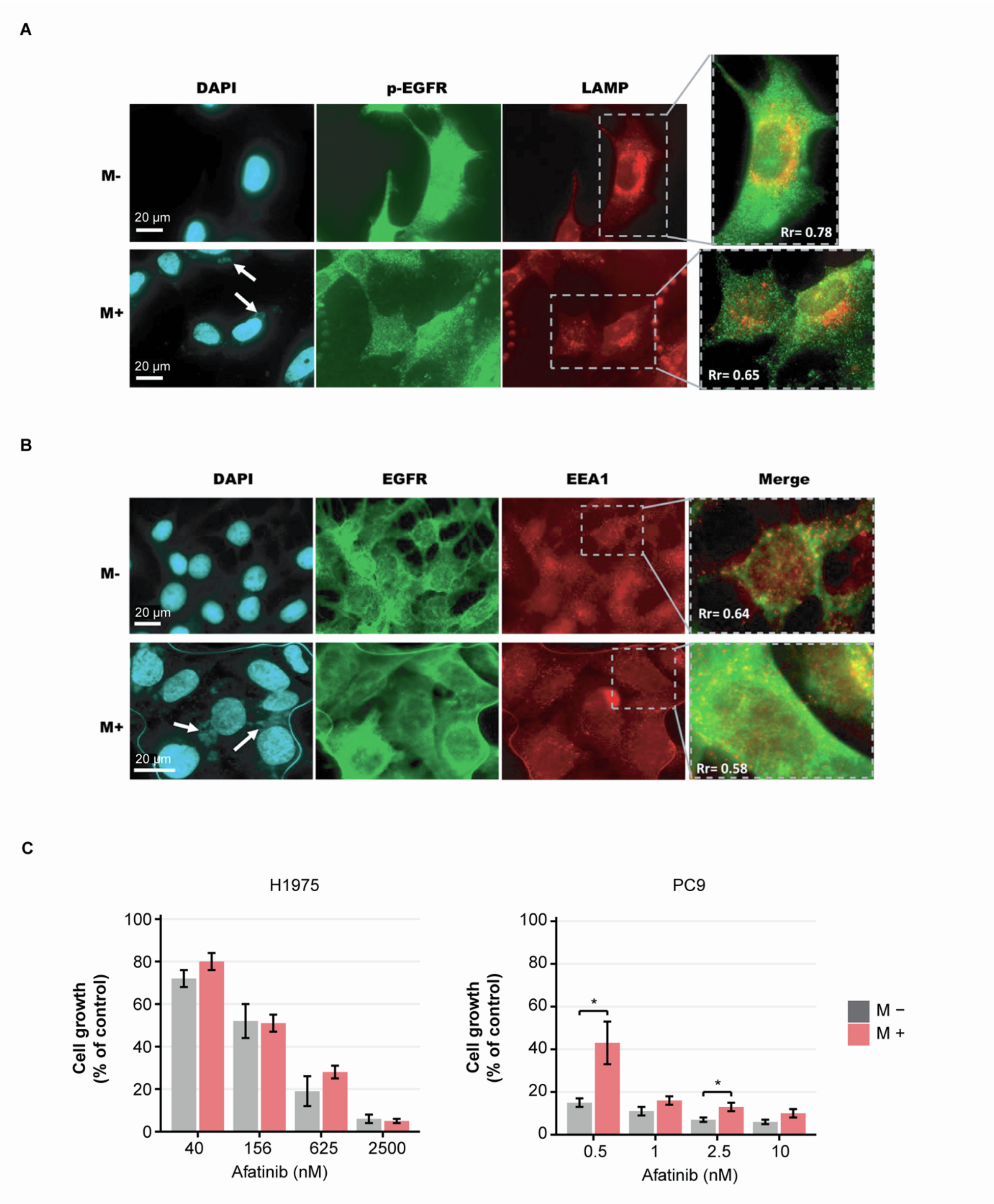

Figure S1. Mycoplasma infection disrupts EGFR trafficking and reinforces drug resistance.
(A) Immunofluorescence co-localization of phospho-EGFR (p-EGFR, green) with lysosomal marker LAMP-3 (red) in H1975 cells. DAPI (cyan) marks nuclei, white arrows indicate mycoplasma aggregates in mycoplasma-positive (M+) cells; Pearson's coefficients are shown as Rr. (B) Co-localization of total EGFR (green) with early endosome marker EEA1 (red) in H1975 cells. White arrows indicate *Mycoplasma* in M+ cells; scale bars, 20 µm. (C) Growth inhibition assays show reduced afatinib sensitivity in M+ cells compared with mycoplasma-negative (M-) cells. All data are presented as means ± SEM (standard error of the mean); *$p < 0.05$.

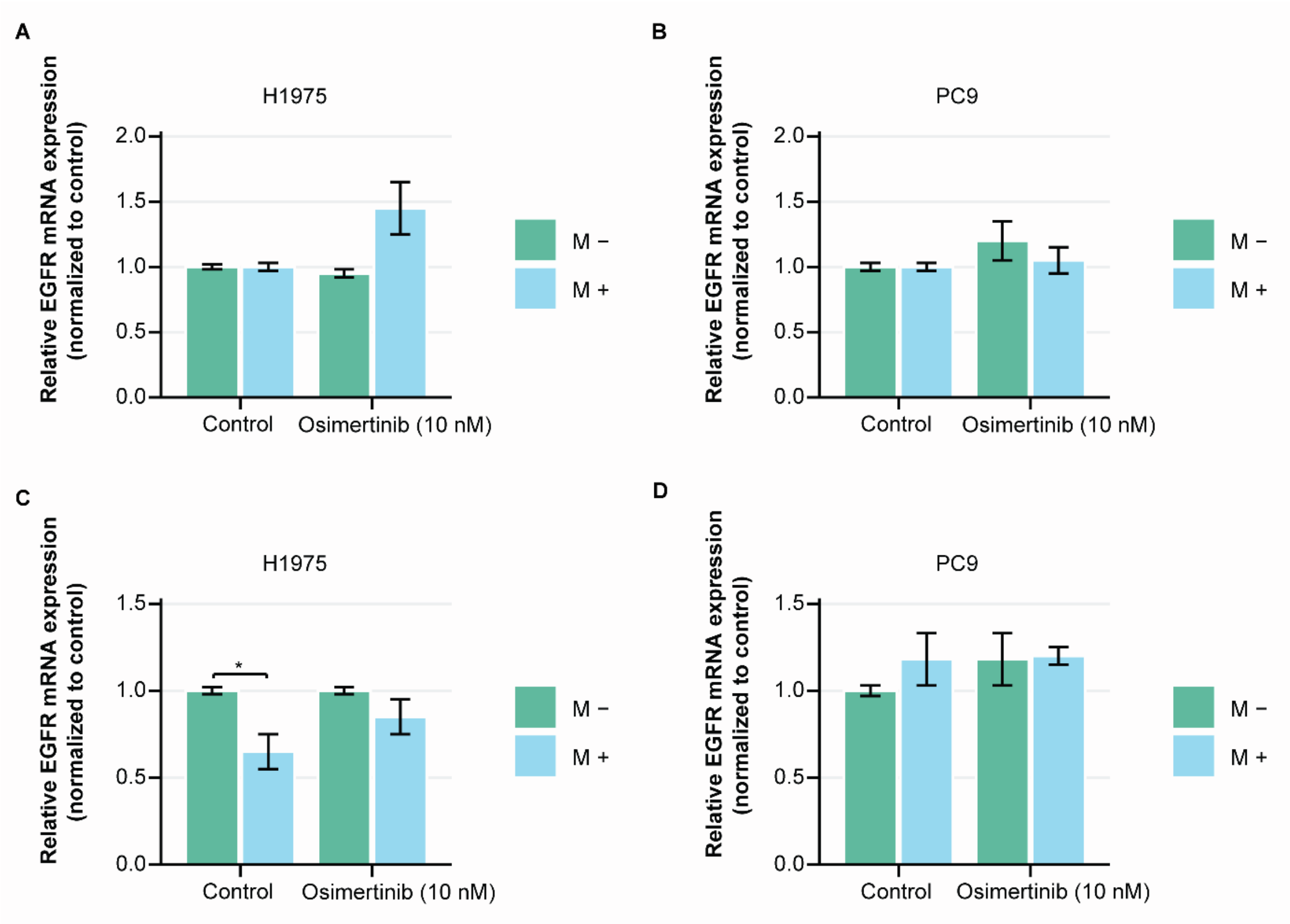

Figure S2. EGFR mRNA expression in H1975 and PC9 cells with or without mycoplasma infection.

RT-qPCR analysis of EGFR mRNA levels in H1975 and PC9 cells under control and osimertinib treatment (10 nM) conditions. Expression was normalized to β-actin. (A-B) Data were corrected to their respective controls (M- to M- control; M+ to M+ control). (C-D) All values were normalized to the M- control. All data are presented as means ± SEM; *$p < 0.05$.

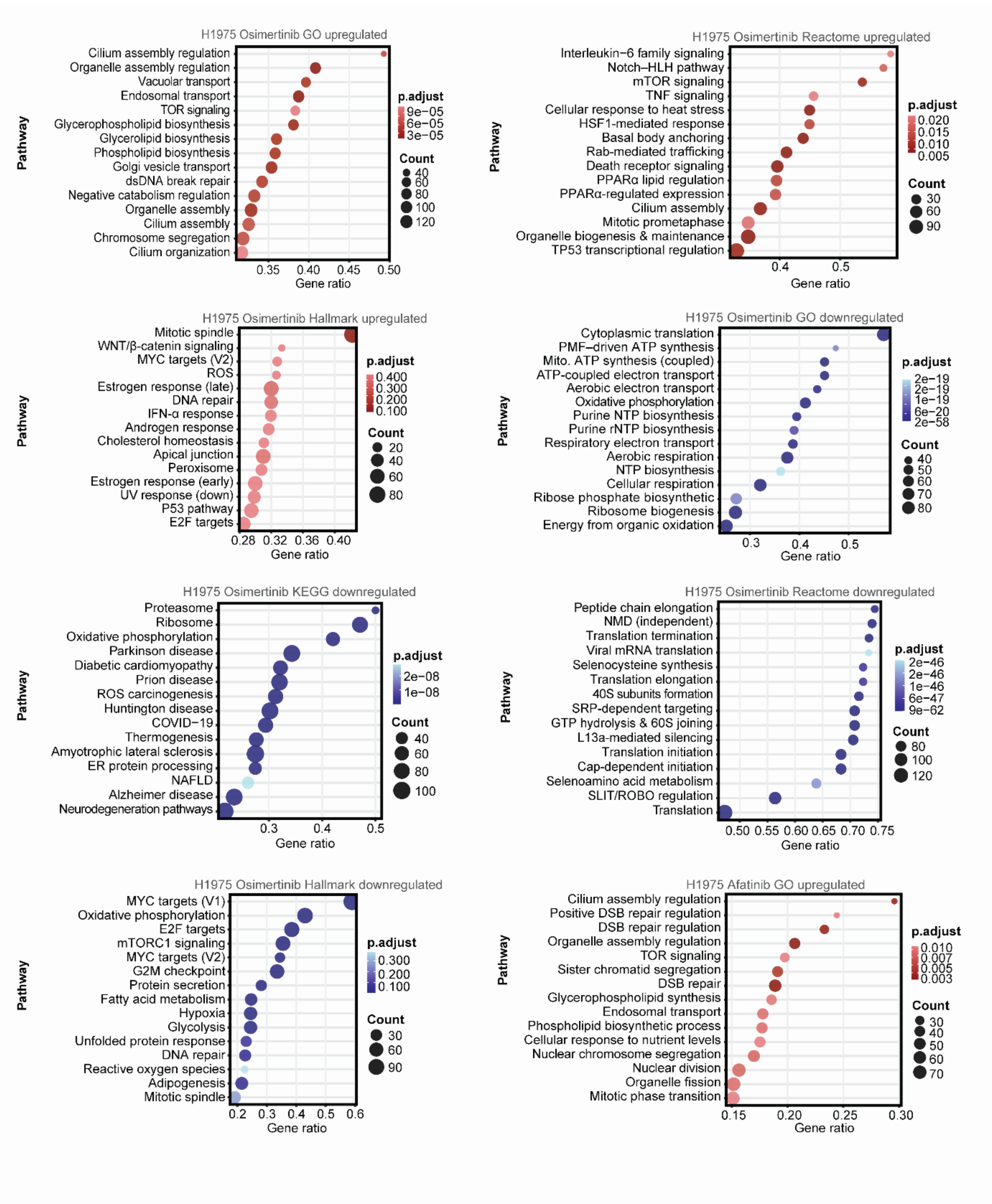
H1975 Osimertinib GO upregulated
Cilium assembly regulation
Organelle assembly regulation
Vacuolar transport
Endosomal transport
TOR signaling
Glycerophospholipid biosynthesis
Glycerolipid biosynthesis
Phospholipid biosynthesis
Golgi vesicle transport
dsDNA break repair
Negative catabolism regulation
Organelle assembly
Cilium assembly
Chromosome segregation
Cilium organization
Pathway
0.35 0.40 0.45 0.50
Gene ratio
p.adjust
9e−05
6e−05
3e−05
Count
40
60
80
100
120
H1975 Osimertinib Reactome upregulated
Interleukin−6 family signaling
Notch−HLH pathway
mTOR signaling
TNF signaling
Cellular response to heat stress
HSF1-mediated response
Basal body anchoring
Rab-mediated trafficking
Death receptor signaling
PPARα lipid regulation
PPARα-regulated expression
Cilium assembly
Mitotic prometaphase
Organelle biogenesis & maintenance
TP53 transcriptional regulation
Pathway
0.4 0.5
Gene ratio
p.adjust
0.020
0.015
0.010
0.005
Count
30
60
90
H1975 Osimertinib Hallmark upregulated
Mitotic spindle
WNT/β-catenin signaling
MYC targets (V2)
ROS
Estrogen response (late)
DNA repair
IFN-α response
Androgen response
Cholesterol homeostasis
Apical junction
Peroxisome
Estrogen response (early)
UV response (down)
P53 pathway
E2F targets
Pathway
0.28 0.32 0.36 0.40
Gene ratio
p.adjust
0.400
0.300
0.200
0.100
Count
20
40
60
80
H1975 Osimertinib GO downregulated
Cytoplasmic translation
PMF−driven ATP synthesis
Mito. ATP synthesis (coupled)
ATP-coupled electron transport
Aerobic electron transport
Oxidative phosphorylation
Purine NTP biosynthesis
Purine rNTP biosynthesis
Respiratory electron transport
Aerobic respiration
NTP biosynthesis
Cellular respiration
Ribose phosphate biosynthetic
Ribosome biogenesis
Energy from organic oxidation
Pathway
0.3 0.4 0.5
Gene ratio
p.adjust
2e−19
2e−19
1e−19
6e−20
2e−58
Count
40
50
60
70
80
H1975 Osimertinib KEGG downregulated
Proteasome
Ribosome
Oxidative phosphorylation
Parkinson disease
Diabetic cardiomyopathy
Prion disease
ROS carcinogenesis
Huntington disease
COVID−19
Thermogenesis
Amyotrophic lateral sclerosis
ER protein processing
NAFLD
Alzheimer disease
Neurodegeneration pathways
Pathway
0.3 0.4 0.5
Gene ratio
p.adjust
2e−08
1e−08
Count
40
60
80
100
H1975 Osimertinib Reactome downregulated
Peptide chain elongation
NMD (independent)
Translation termination
Viral mRNA translation
Selenocysteine synthesis
Translation elongation
40S subunits formation
SRP-dependent targeting
GTP hydrolysis & 60S joining
L13a-mediated silencing
Translation initiation
Cap-dependent initiation
Selenoamino acid metabolism
SLIT/ROBO regulation
Translation
Pathway
0.50 0.55 0.60 0.65 0.70 0.75
Gene ratio
p.adjust
2e−46
2e−46
1e−46
6e−47
9e−62
Count
80
100
120
H1975 Osimertinib Hallmark downregulated
MYC targets (V1)
Oxidative phosphorylation
E2F targets
mTORC1 signaling
MYC targets (V2)
G2M checkpoint
Protein secretion
Fatty acid metabolism
Hypoxia
Glycolysis
Unfolded protein response
DNA repair
Reactive oxygen species
Adipogenesis
Mitotic spindle
Pathway
0.2 0.3 0.4 0.5 0.6
Gene ratio
p.adjust
0.300
0.200
0.100
Count
30
60
90
H1975 Afatinib GO upregulated
Cilium assembly regulation
Positive DSB repair regulation
DSB repair regulation
Organelle assembly regulation
TOR signaling
Sister chromatid segregation
DSB repair
Glycerophospholipid synthesis
Endosomal transport
Phospholipid biosynthetic process
Cellular response to nutrient levels
Nuclear chromosome segregation
Nuclear division
Organelle fission
Mitotic phase transition
Pathway
0.15 0.20 0.25 0.30
Gene ratio
p.adjust
0.010
0.007
0.005
0.003
Count
30
40
50
60
70

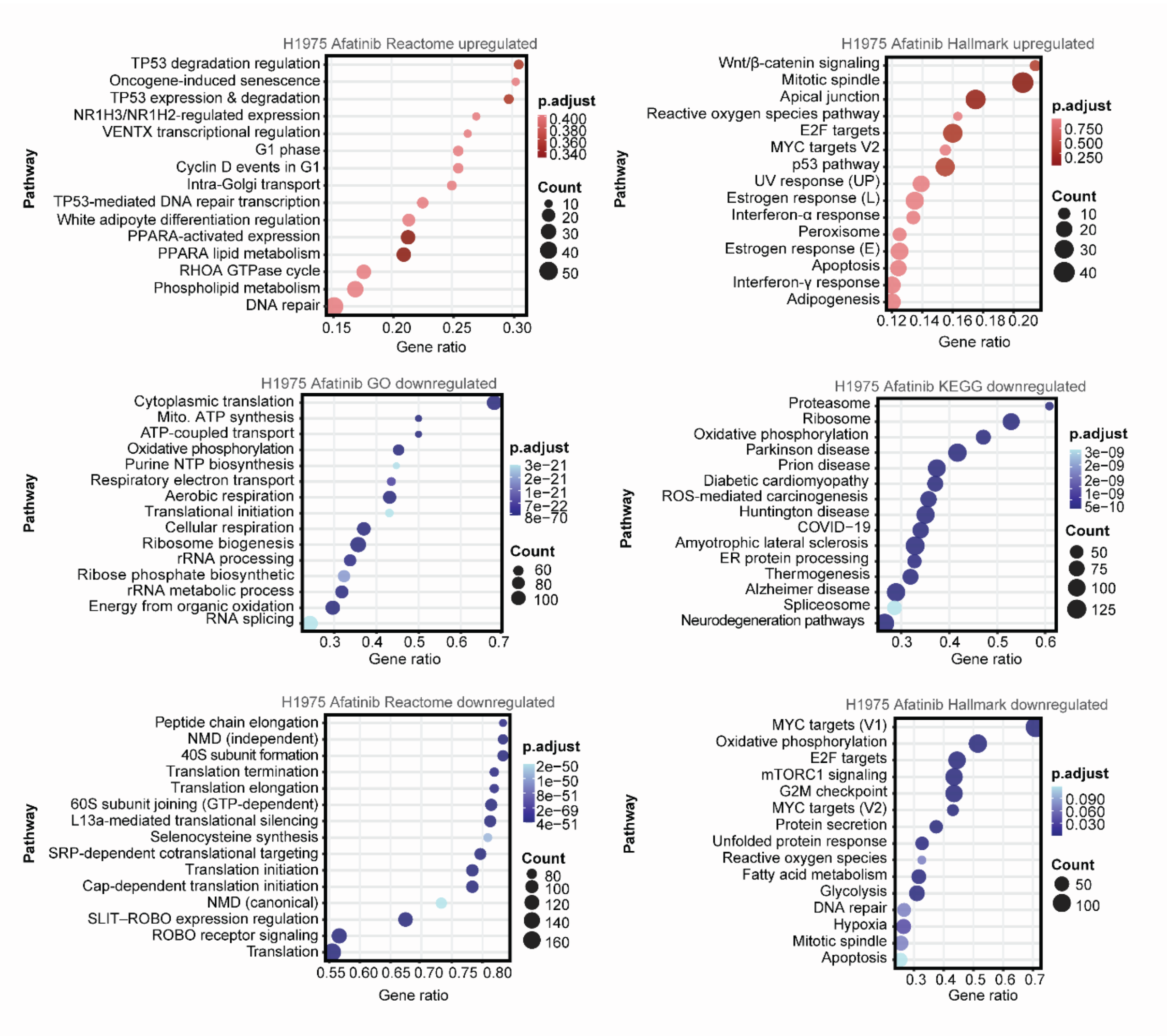

H1975 Afatinib Reactome upregulated
TP53 degradation regulation
Oncogene-induced senescence
TP53 expression & degradation
NR1H3/NR1H2-regulated expression
VENTX transcriptional regulation
G1 phase
Cyclin D events in G1
Intra-Golgi transport
TP53-mediated DNA repair transcription
White adipoyte differentiation regulation
PPARA-activated expression
PPARA lipid metabolism
RHOA GTPase cycle
Phospholipid metabolism
DNA repair
Pathway
Gene ratio
p.adjust
Count
H1975 Afatinib Hallmark upregulated
Wnt/β-catenin signaling
Mitotic spindle
Apical junction
Reactive oxygen species pathway
E2F targets
MYC targets V2
p53 pathway
UV response (UP)
Estrogen response (L)
Interferon-α response
Peroxisome
Estrogen response (E)
Apoptosis
Interferon-γ response
Adipogenesis
H1975 Afatinib GO downregulated
Cytoplasmic translation
Mito. ATP synthesis
ATP-coupled transport
Oxidative phosphorylation
Purine NTP biosynthesis
Respiratory electron transport
Aerobic respiration
Translational initiation
Cellular respiration
Ribosome biogenesis
rRNA processing
Ribose phosphate biosynthetic
rRNA metabolic process
Energy from organic oxidation
RNA splicing
H1975 Afatinib KEGG downregulated
Proteasome
Ribosome
Oxidative phosphorylation
Parkinson disease
Prion disease
Diabetic cardiomyopathy
ROS-mediated carcinogenesis
Huntington disease
COVID−19
Amyotrophic lateral sclerosis
ER protein processing
Thermogenesis
Alzheimer disease
Spliceosome
Neurodegeneration pathways
H1975 Afatinib Reactome downregulated
Peptide chain elongation
NMD (independent)
40S subunit formation
Translation termination
Translation elongation
60S subunit joining (GTP-dependent)
L13a-mediated translational silencing
Selenocysteine synthesis
SRP-dependent cotranslational targeting
Translation initiation
Cap-dependent translation initiation
NMD (canonical)
SLIT–ROBO expression regulation
ROBO receptor signaling
Translation
H1975 Afatinib Hallmark downregulated
MYC targets (V1)
Oxidative phosphorylation
E2F targets
mTORC1 signaling
G2M checkpoint
MYC targets (V2)
Protein secretion
Unfolded protein response
Reactive oxygen species
Fatty acid metabolism
Glycolysis
DNA repair
Hypoxia
Mitotic spindle
Apoptosis

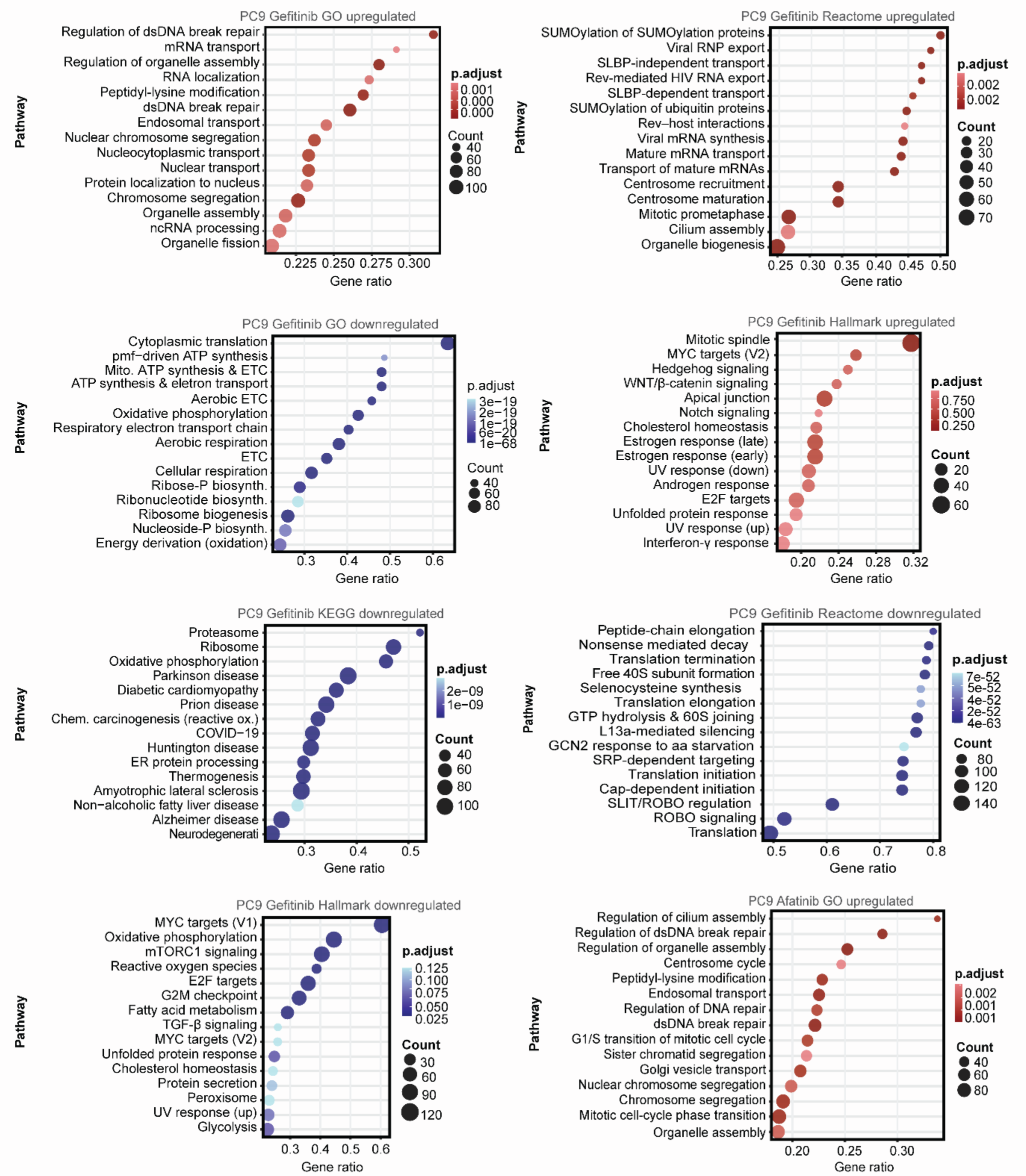

PC9 Gefitinib GO upregulated
Regulation of dsDNA break repair
mRNA transport
Regulation of organelle assembly
RNA localization
Peptidyl-lysine modification
dsDNA break repair
Endosomal transport
Nuclear chromosome segregation
Nucleocytoplasmic transport
Nuclear transport
Protein localization to nucleus
Chromosome segregation
Organelle assembly
ncRNA processing
Organelle fission
0.225 0.250 0.275 0.300
Gene ratio
Pathway
p.adjust
0.001
0.000
0.000
Count
40
60
80
100
PC9 Gefitinib Reactome upregulated
SUMOylation of SUMOylation proteins
Viral RNP export
SLBP-independent transport
Rev-mediated HIV RNA export
SLBP-dependent transport
SUMOylation of ubiquitin proteins
Rev–host interactions
Viral mRNA synthesis
Mature mRNA transport
Transport of mature mRNAs
Centrosome recruitment
Centrosome maturation
Mitotic prometaphase
Cilium assembly
Organelle biogenesis
0.25 0.30 0.35 0.40 0.45 0.50
Gene ratio
Pathway
p.adjust
0.002
0.002
Count
20
30
40
50
60
70
PC9 Gefitinib GO downregulated
Cytoplasmic translation
pmf−driven ATP synthesis
Mito. ATP synthesis & ETC
ATP synthesis & eletron transport
Aerobic ETC
Oxidative phosphorylation
Respiratory electron transport chain
Aerobic respiration
ETC
Cellular respiration
Ribose-P biosynth.
Ribonucleotide biosynth.
Ribosome biogenesis
Nucleoside-P biosynth.
Energy derivation (oxidation)
0.3 0.4 0.5 0.6
Gene ratio
Pathway
p.adjust
3e−19
2e−19
1e−19
6e−20
1e−68
Count
40
60
80
PC9 Gefitinib Hallmark upregulated
Mitotic spindle
MYC targets (V2)
Hedgehog signaling
WNT/β-catenin signaling
Apical junction
Notch signaling
Cholesterol homeostasis
Estrogen response (late)
Estrogen response (early)
UV response (down)
Androgen response
E2F targets
Unfolded protein response
UV response (up)
Interferon-γ response
0.20 0.24 0.28 0.32
Gene ratio
Pathway
p.adjust
0.750
0.500
0.250
Count
20
40
60
PC9 Gefitinib KEGG downregulated
Proteasome
Ribosome
Oxidative phosphorylation
Parkinson disease
Diabetic cardiomyopathy
Prion disease
Chem. carcinogenesis (reactive ox.)
COVID−19
Huntington disease
ER protein processing
Thermogenesis
Amyotrophic lateral sclerosis
Non−alcoholic fatty liver disease
Alzheimer disease
Neurodegenerati
0.3 0.4 0.5
Gene ratio
Pathway
p.adjust
2e−09
1e−09
Count
40
60
80
100
PC9 Gefitinib Reactome downregulated
Peptide-chain elongation
Nonsense mediated decay
Translation termination
Free 40S subunit formation
Selenocysteine synthesis
Translation elongation
GTP hydrolysis & 60S joining
L13a-mediated silencing
GCN2 response to aa starvation
SRP-dependent targeting
Translation initiation
Cap-dependent initiation
SLIT/ROBO regulation
ROBO signaling
Translation
0.5 0.6 0.7 0.8
Gene ratio
Pathway
p.adjust
7e-52
5e-52
4e-52
2e-52
4e-63
Count
80
100
120
140
PC9 Gefitinib Hallmark downregulated
MYC targets (V1)
Oxidative phosphorylation
mTORC1 signaling
Reactive oxygen species
E2F targets
G2M checkpoint
Fatty acid metabolism
TGF-β signaling
MYC targets (V2)
Unfolded protein response
Cholesterol homeostasis
Protein secretion
Peroxisome
UV response (up)
Glycolysis
0.3 0.4 0.5 0.6
Gene ratio
Pathway
p.adjust
0.125
0.100
0.075
0.050
0.025
Count
30
60
90
120
PC9 Afatinib GO upregulated
Regulation of cilium assembly
Regulation of dsDNA break repair
Regulation of organelle assembly
Centrosome cycle
Peptidyl-lysine modification
Endosomal transport
Regulation of DNA repair
dsDNA break repair
G1/S transition of mitotic cell cycle
Sister chromatid segregation
Golgi vesicle transport
Nuclear chromosome segregation
Chromosome segregation
Mitotic cell-cycle phase transition
Organelle assembly
0.20 0.25 0.30
Gene ratio
Pathway
p.adjust
0.002
0.001
0.001
Count
40
60
80

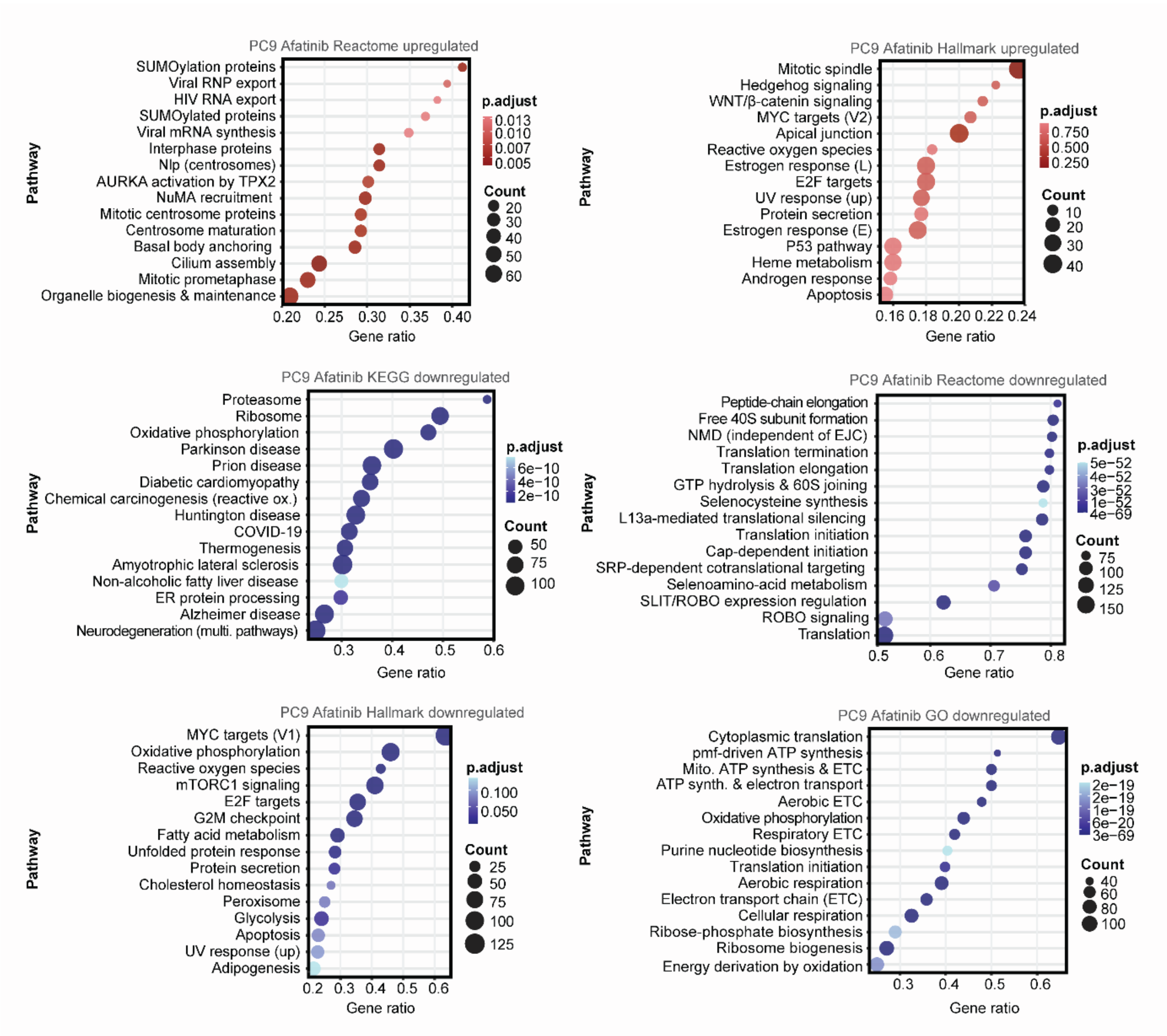

Figure S3. Transcriptomic pathway enrichment in H1975 and PC9 models.

Differentially expressed genes from H1975 and PC9 cells (±TKIs) were analyzed using GO, KEGG, Reactome and Hallmark gene sets. Separate panels show upregulated (red) and downregulated pathways (blue).

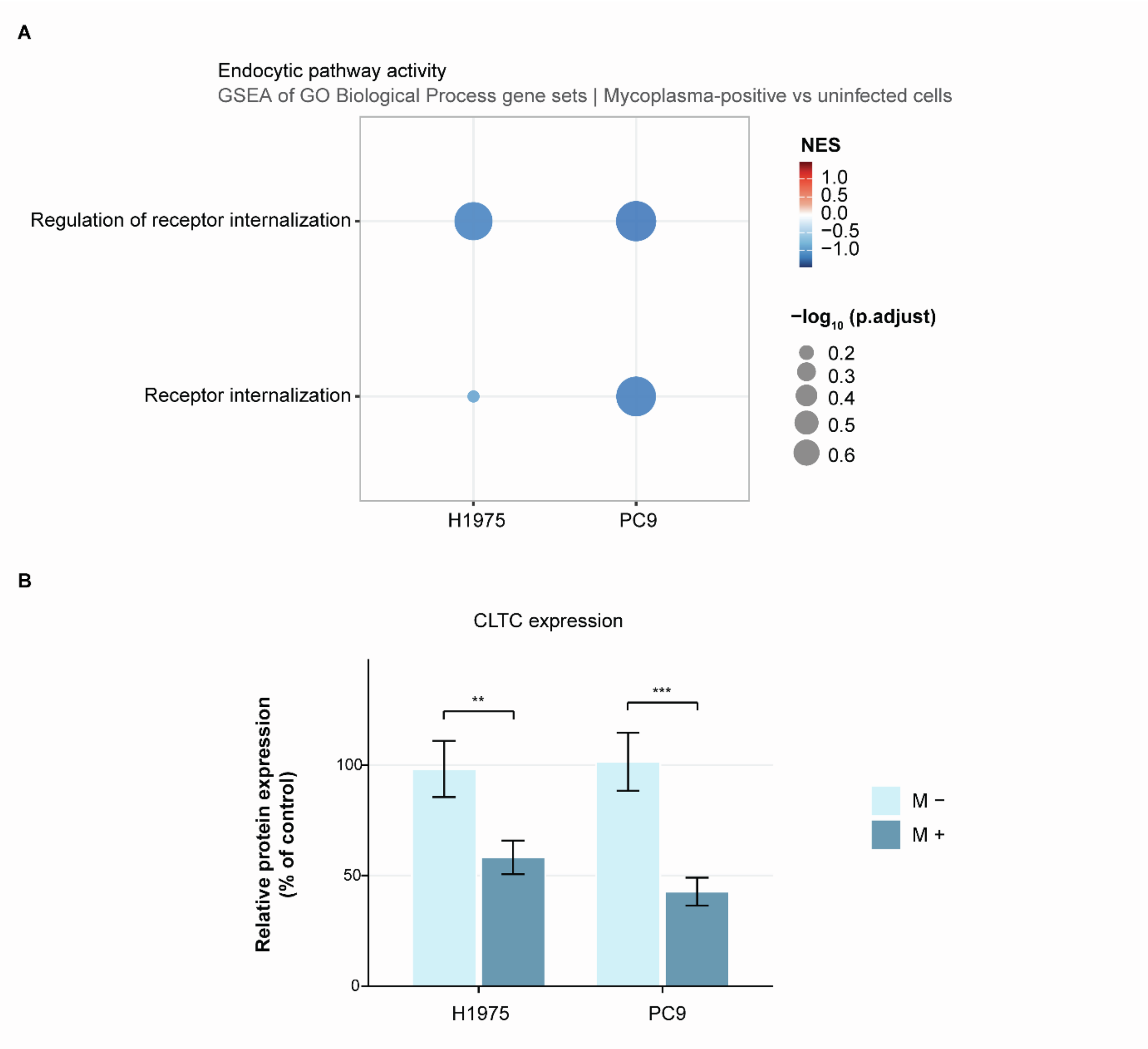

Figure S4. Endocytic regulator expression in mycoplasma-infected cells.

(A) GSEA bubble plot showing enrichment of GO Biological Process gene sets related to receptor internalization in M+ versus M- H1975 and PC9 cells. Gene sets shown: “Regulation of Receptor Internalization” (GO:0002090) and “Receptor Internalization” (GO:0031623). Bubble size represents -$\log_{10}$(adjusted *p* value), color represents normalized enrichment score (NES). (B) Enzyme-Linked Immunosorbent Assay (ELISA) of clathrin in M+ and M- H1975 and PC9 cells. Data are presented as means ± SEM; $^{**}p < 0.01$, $^{***}p < 0.001$.

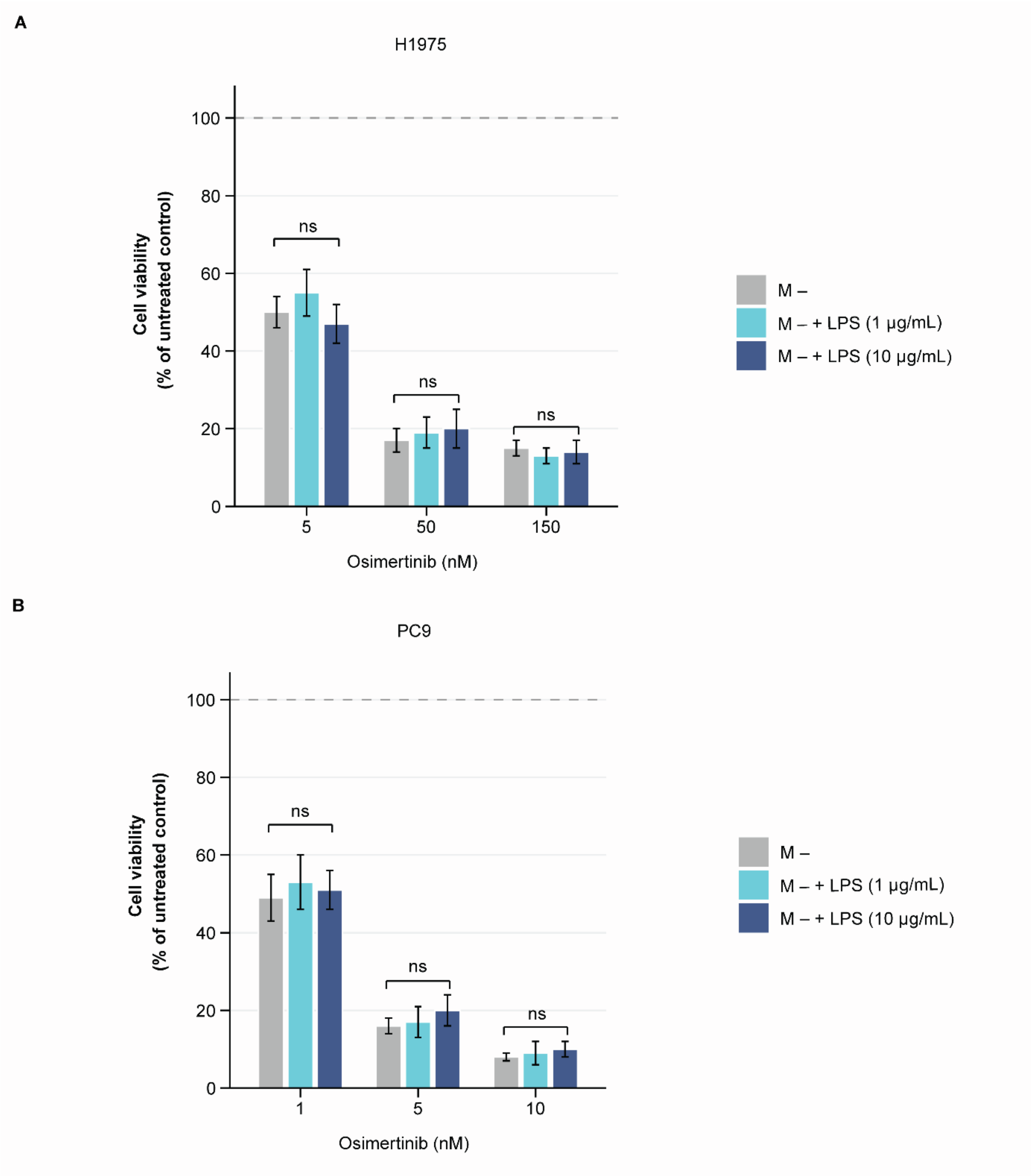

Figure S5. LPS stimulation does not reproduce mycoplasma-mediated osimertinib resistance. (A-B) Cell viability of mycoplasma-negative H1975 (A) and PC9 (B) cells following treatment with osimertinib (nM) in the presence or absence of lipopolysaccharide (LPS; 1 or 10 μg/mL). Data are presented as mean ± SEM; ns, not significant.

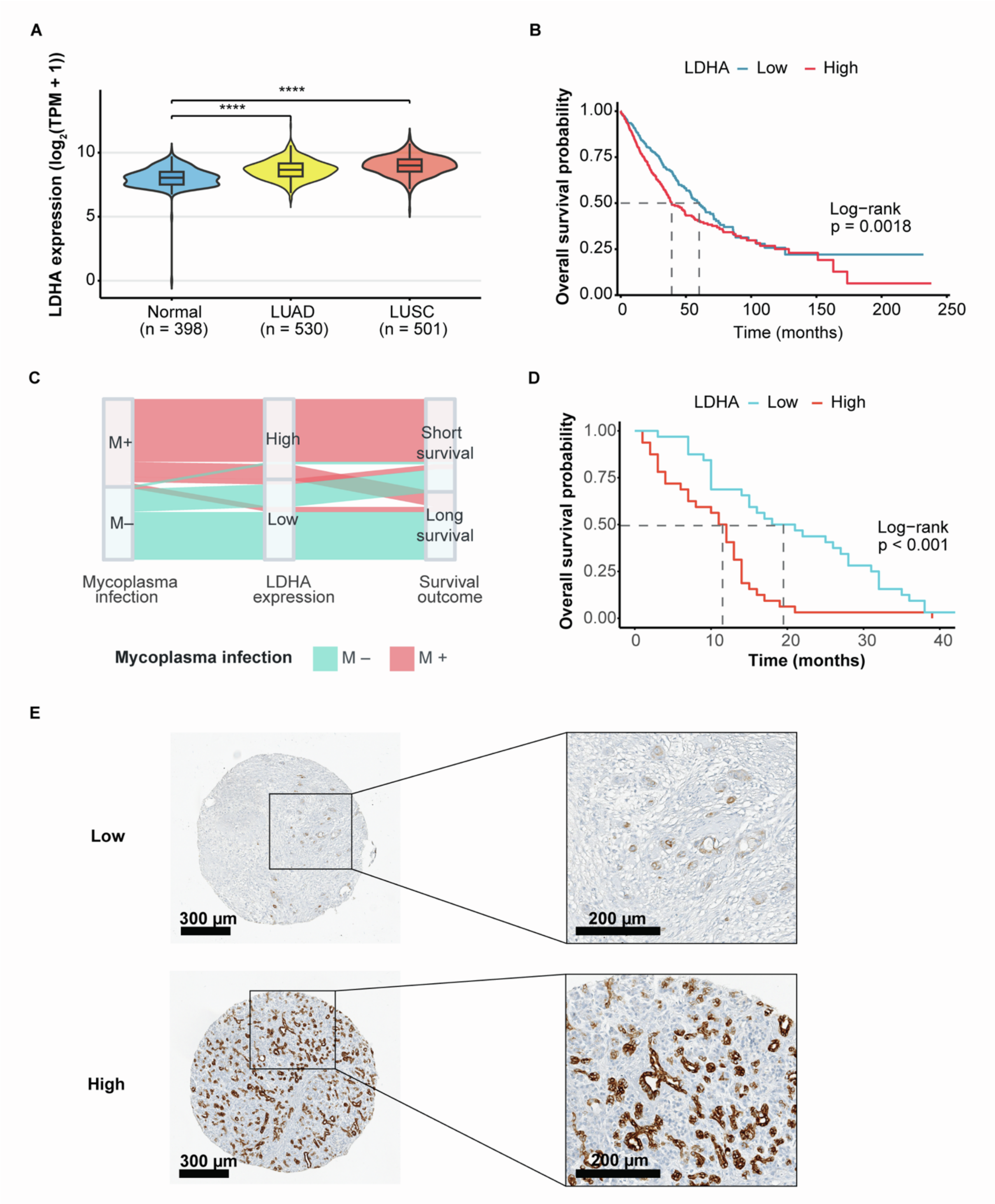

Figure S6. LDHA expression correlates with mycoplasma infection status and predicts survival outcomes in lung cancer patients.

(A) LDHA mRNA expression levels in normal lung tissue (n=398) compared to lung adenocarcinoma (LUAD, n=530) and lung squamous cell carcinoma (LUSC, n=501) from TCGA and GTEx databases (****p < 0.0001, Wilcoxon rank-sum test). (B) Overall survival by LDHA expression (median split) in TCGA-GTEx lung cancer cohort (log-rank p = 0.0018). Dashed lines indicate median survival times for each group. (C) Sankey diagram illustrating the relationship among mycoplasma infection status, LDHA expression level, and survival outcome in 64 EGFR-TKI-treated NSCLC patients. (D) Kaplan-Meier

analysis of overall survival in an independent patient cohort stratified by LDHA expression. High LDHA expression is associated with poorer survival (log-rank $p < 0.001$). (E) Representative immunohistochemistry of LDHA expression in lung cancer tissue showing low (upper) and high (lower) expression patterns. Scale bars as indicated.

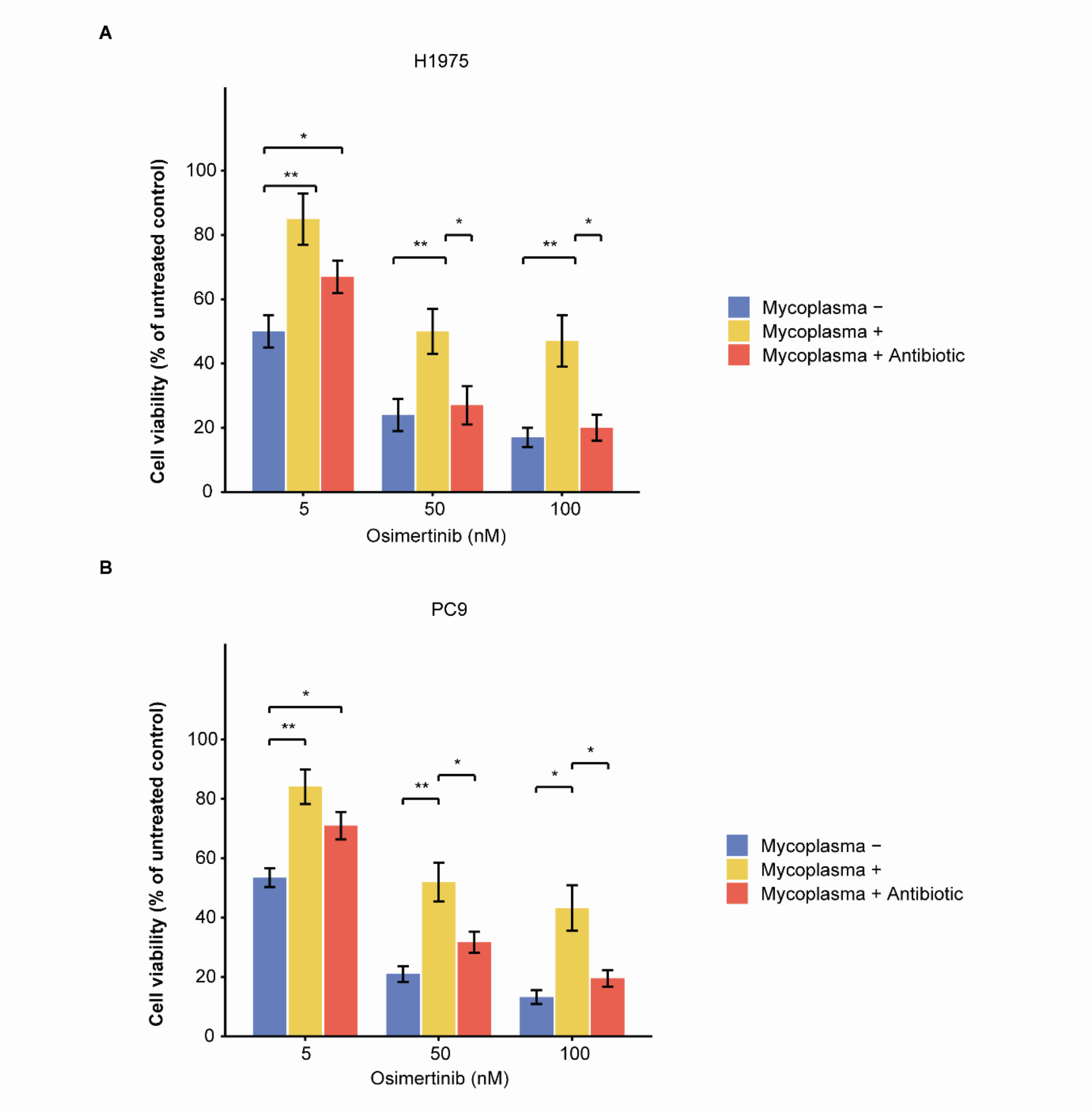

Figure S7. Antibiotic eradication of *Mycoplasma* restores sensitivity to osimertinib in EGFR-mutant NSCLC cells.

(A-B) Cell viability of H1975 (A) and PC9 (B) cells following treatment with increasing concentrations of osimertinib (5, 50, and 100 nM) under three conditions: mycoplasma-negative, mycoplasma-positive, and mycoplasma-positive following antibiotic treatment. Antibiotic-mediated mycoplasma clearance partially restored osimertinib sensitivity at all concentrations. Data are presented as means ± SEM; *$p < 0.05$, **$p < 0.01$.

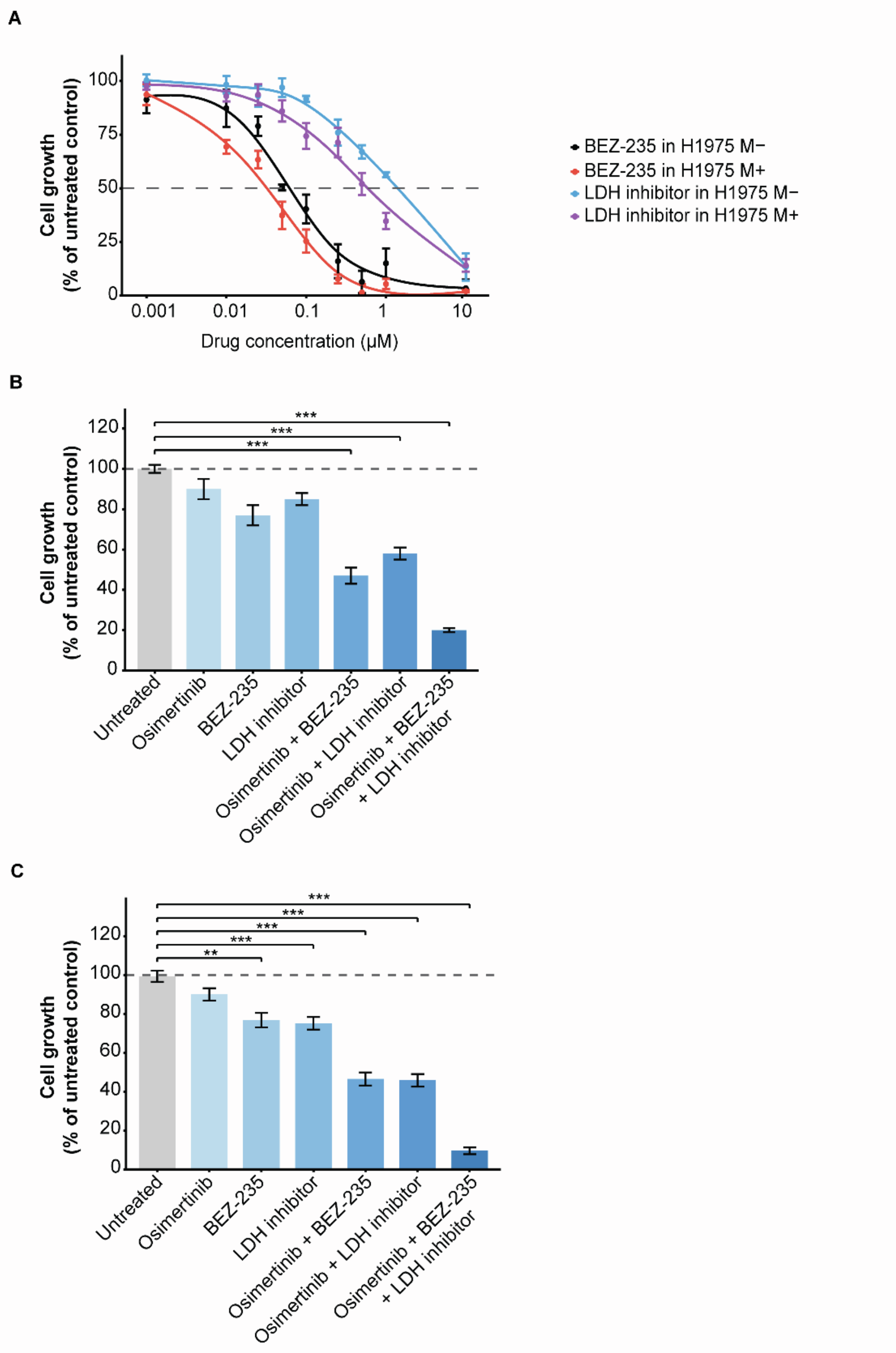

Figure S8. Mycoplasma infection reduces drug sensitivity and enhances the therapeutic effect of combined pathway inhibition.

(A) Concentration-response curves of BEZ-235 and LDHA inhibitor in H1975 cells with (M+) or (M-) without mycoplasma infection. Data represent mean ± SEM. The dashed line indicates 50% viability. (B-C) Cell viability following single or combination drug treatment in mycoplasma-infected H1975 (B) and PC9 (C) cells. Data represent mean ± SEM; ***p* < 0.01, ****p* < 0.001 vs. untreated control (one-way ANOVA).

Table S1. Clinicopathological characteristics of EGFR-TKI-treated NSCLC patients and association with overall survival (OS)

Characteristics	Patients, n (%)	Median OS, months	P-value
Sex			0.595
Female	32 (50.0)	14.0	
Male	32 (50.0)	10.5	
Stage			0.435
I-III	27 (42.2)	11.0	
IV	37 (57.8)	14.0	
Smoking history			0.775
Yes	40 (62.5)	13.5	
No	24 (37.5)	13.0	
Histological type			<0.001
Adenocarcinoma	58 (90.6)	14.0	
Squamous cell carcinoma	6 (9.4)	7.0	
Mycoplasma infection			**<0.001**
Positive	35 (54.7)	12.0	
Negative	29 (45.3)	22.0	
LDHA expression			**<0.001**
High	32 (50.0)	11.5	
Low	32 (50.0)	19.5	

Notes. This table presents the baseline clinicopathological characteristics of 64 EGFR-TKI-treated NSCLC patients. Patient characteristics include sex, tumor stage, smoking history, histological type, mycoplasma infection status, and LDHA expression. Median overall survival (OS) was estimated using the Kaplan-Meier method. *P* values were calculated using the log-rank test. Statistically significant associations ($p < 0.05$) are shown in bold. OS, overall survival.
